# Evaluating a Peer-Support Mobile App for Mental Health and Substance Use Among Adolescents Over 12 Months During the COVID-19 Pandemic: Randomized Controlled Trial

**DOI:** 10.2196/45216

**Published:** 2023-09-27

**Authors:** Louise Birrell, Jennifer Debenham, Ainsley Furneaux-Bate, Katrina Prior, Sophia Spallek, Louise Thornton, Catherine Chapman, Nicola Newton

**Affiliations:** 1 The Matilda Centre for Research in Mental Health and Substance Use The University of Sydney Sydney Australia

**Keywords:** mental health, substance use, prevention, school-based, peer support, anxiety, social support, psychosocial support systems, depression, adolescent, mobile apps, eHealth, mHealth, mobile phone, COVID-19

## Abstract

**Background:**

Although it is well known that adolescents frequently turn to their friends for support around mental health and substance use problems, there are currently no evidence-based digital programs to support them to do this.

**Objective:**

The aim of this study was to evaluate the efficacy of the *Mind your Mate* program, a digital peer-support program, in improving mental health symptoms, reducing the uptake of substance use, and increasing help seeking. The *Mind your Mate* program consists of a 40-minute web-based classroom lesson and a companion smartphone mobile app. The active control group received school-based health education as usual.

**Methods:**

A cluster randomized controlled trial was conducted with 12 secondary schools and 166 students (mean age 15.3, SD 0.41 years; 72/166, 43.4% female; and 133/166, 80.1% born in Australia). Participants completed self-reported questionnaires assessing symptoms of mental health (depression, anxiety, and psychological distress), substance use (alcohol and other drug use), and help-seeking measures at baseline and at 6-month and 12-month follow-ups.

**Results:**

Students who received the *Mind your Mate* program had greater reductions in depressive symptoms over a 12-month period than controls (b=−1.86, 95% CI −3.73 to 0.02; Cohen *d=−*0.31). Anxiety symptoms decreased among students in the intervention group; however, these reductions did not meet statistical significance thresholds. No differences were observed in relation to psychological distress or help-seeking.

**Conclusions:**

Small to moderate reductions in depression symptoms were observed among students allocated to receive the *Mind your Mate* intervention. Although the current results are encouraging, there is a need to continue to refine, develop, and evaluate innovative applied approaches for the prevention of mental disorders in real-world settings.

**Trial Registration:**

Australian New Zealand Clinical Trials Registry (ANZCTR) ACTRN12620000753954; https://anzctr.org.au/Trial/Registration/TrialReview.aspx?ACTRN=12620000753954

**International Registered Report Identifier (IRRID):**

RR2-10.2196/26796

## Introduction

### Background

Youth mental health has been identified as a global priority. It is estimated that the global prevalence of mental disorders among adolescents and children is almost 14% [1], with mental disorders being the largest contributor to the global burden of disease in terms of years lived with disability [2]. They result in significant impact on functioning, schooling, health, and well-being with huge economic costs [3]. Concerningly, common disorders, such as depression and anxiety, are on the rise among recent cohorts of young people [4], with the COVID-19 pandemic worsening the impact [5]. Many young people also continue to use and misuse alcohol and other drugs [6], with mental disorders being a substantive risk factor for the development of problematic substance use [7].

The onset of mental and substance use disorders typically occurs between adolescence and early adulthood; however, most young people do not seek professional help [8]. Rather, adolescents commonly turn to their friends when experiencing mental health problems [9] and, more generally, when facing a significant issue in their lives [10]. In an extensive survey of >6500 young people, friends were the most commonly cited source of help for young people generally and for young people experiencing high levels of psychological distress [11]. Interventions promoting social support and connection have been identified as an important target for mental health prevention [12]. However, it is not clear whether young people are adequately equipped to provide evidence-based and effective social support to their peers in relation to mental and substance use disorders. A systematic review of existing school-based peer interventions showed that despite the widespread use of peer interventions, there is a lack of evidence demonstrating mental health improvements [13]. There is a clear need to explore novel prevention initiatives that provide evidence-based tools that young people can access to support their peers. Such programs must be easily accessible, engaging, and meeting young people where they are.

To be engaging and effective, interventions should be delivered in modes relevant to adolescents, considering the unequivocal access to digital technologies that recent cohorts of adolescents have. In high-income countries, such as the United States, the United Kingdom, and Australia, 90% to 95% of teenagers own a mobile phone, nearly all of which are internet-connected smartphones [14-16]. One key opportunity for the prevention of mental disorders is harnessing mobile and web-based technology to deliver easily accessible prevention that can be widely disseminated at a relatively low cost, in a format that is acceptable and engaging to adolescents. The COVID-19 pandemic initiated a paradigm shift, accelerating the uptake of digital technologies to deliver scalable, accessible, and affordable mental health care [17]. Although mental health apps and web-based programs have proliferated, there is a lack of evidence-based interventions aiming to upskill adolescents around supporting peers in relation to mental health and substance use.

The *Mind your Mate* program, a classroom lesson and mobile app, aims to upskill and empower adolescents to better support their peers. Adolescents completed 1 web-based lesson in class that introduces the concept of supporting peers and mental health and substance use literacy. Students were prompted to download and register on the *Mind your Mate* smartphone app at the end of the lesson. The app is a self-guided program that builds upon the concepts introduced in the classroom lesson by providing normative information about mental health and substance use, skill-building modules for peer support (including active listening and checking in), self-care modules, as well as referral and support options to encourage further help seeking. Full details regarding the app development process, app features, and functionality have been previously published [18,19]. The next step was to evaluate the effectiveness of the program in improving youth mental health.

### Aims

The aim of this study was to evaluate the efficacy of the *Mind your Mate* program through a cluster randomized controlled trial (RCT) conducted in partnership with Australian secondary schools. As specified in our preregistered protocol, we hypothesized that the *Mind your Mate* intervention would be more effective than active control over a 12-month period in (1) delaying the uptake of alcohol and other drugs; (2) reducing anxiety and depression symptoms; (3) increasing knowledge about mental health, alcohol, and other drugs; and (4) increasing help-seeking behavior.

## Methods

### Ethics Approval

This study was approved by the University of Sydney Human Research Ethics Committee, Australia (project number: 2020/054), and prospectively registered in the Australian New Zealand Clinical Trials Registry (ACTRN 12620000753954).

### Access to Schools

Access to schools was granted by the New South Wales (NSW) Department of Education and Communities State Education Research Applications Process (2020130).

### Study Design

A cluster RCT was conducted in 12 independent and public secondary schools in the Greater Sydney region of NSW. Schools were randomly allocated to 1 of the 2 parallel arms: the *Mind your Mate* condition or the active control condition (health education as usual). Cluster randomization was used to avoid contamination of the control condition by the intervention through student communication. All year 9 students (approximately aged 14 years) at the participating schools were invited to participate. The full study protocol including the initial sample size calculations was prospectively published, and it was estimated that 12 schools with at least 720 students would be required to achieve 80% power to detect an effect size of 0.2 [18]. Student participation rates did not meet the sample size targets, largely because of recruitment occurring during the first wave of the unfolding COVID-19 pandemic, characterized by school closures and uncertainty, meaning that parental consent was difficult to obtain. Given that we did not achieve the intended sample size (resulting in reduced power to detect significant effects), we have now focused on the magnitude of the effects found rather than on the statistical significance.

### Recruitment

Recruitment took place between June 2020 and March 2021. A random selection of 126 independent and public schools located within the Greater Sydney area of NSW were contacted using publicly available details. This study was also advertised through the researchers’ networks.

School principals and relevant school staff members (eg, Head of Wellbeing) were initially approached using an invitation email outlining the aims of the study and inviting their school to take part. The research team followed up with the staff via phone calls and emails. Principals who agreed for their school to participate were asked to identify an appropriate staff member (eg, head of student well-being or health and physical education coordinator) who became the point of contact for study coordination.

Opt-in parental and guardian information and consent forms were sent electronically or via hard copy to all parents or guardians of the selected year group. Students were eligible to participate in the trial if they received parental consent, provided active consent, and owned a smartphone.

### Randomization

Randomization of schools was conducted by a biostatistician external to the research team using the *block-rand* function in R (R Foundation for Statistical Computing). Schools were stratified according to gender, with the school gender mix consisting of coeducational, predominately male, or predominately female students. The threshold for predominately male or predominately female students was at >60%. Half of the schools were randomly allocated to the *Mind your Mate* intervention condition (n=6) and half were allocated to the active control condition who received health education as usual (n=6). Teachers, students, and researchers were not blinded to the intervention allocation.

### Intervention Group

The *Mind your Mate* intervention aimed to upskill and empower adolescents to better support their peers and encourage help seeking. It was designed in collaboration with young people and experts in mental health and substance use prevention. Key content and module selection was based on a scoping review of the literature, a survey and focus groups with young people, and evidence-based mental health and substance use prevention strategies (full details of the development process have been previously published [19]). The intervention included 1 introductory web-based classroom lesson, led by a teacher, and a self-directed mobile app.

### Classroom Lesson

The *Mind your Mate* classroom lesson is a web-based educational resource facilitated by teachers at the participating schools and delivered through the study website. Teachers were given an implementation guide with instructions for students to access the classroom lesson and an outline of the links to the NSW Personal Development, Health, and Physical Education Syllabus. The lesson lasted for approximately 40 minutes and contained animated examples, quizzes, and both web-based and classroom activities, introducing the concept of supporting peers, mental health and substance use literacy, help seeking, and harm-minimization information in relation to substance use. At the completion of the lesson, a video introduced the *Mind your Mate* app, demonstrating its features and directing students to download the app onto their mobile phone. Students were asked to use the mobile app as needed in a self-directed manner over the following 12 months.

### Mobile App

The *Mind your Mate* mobile app was designed to facilitate peer support and help seeking and to provide key mental health literacy information about anxiety, depression, and substance use. The app provided tools to help facilitate communication between peers, including templates and examples of these conversations. It contained 10 educational modules on mental health and substance use, as well as the functionality to schedule conversations with peers, a mood tracker, and a self-care activity scheduling feature (further details on the app content can be found in the study by Birrell et al [18]). The app was designed to be self-guided and was only accessible to students attending schools allocated to the intervention group. A school-specific code was required to access the app. Throughout the app use, it was emphasized that students were not responsible for their friends and their friends’ choices and were encouraged to connect their peers with a responsible adult. Crisis helpline numbers were easily accessible on all screens and under “support options.”

### Active Control Group

Schools allocated to the control condition were asked to implement their usual health and physical education classes during the 12-month trial period. The year 9 and 10 health and physical education curriculum mandates that well-being and alcohol and other drug use content be taught. As such, all control schools delivered curriculum-based health education during the trial, ranging from 1 to 15 mental health lessons and from 2 to 21 lessons on alcohol and other drugs. Control schools were offered access to the intervention at the completion of the trial.

### Procedure

Baseline data collection was conducted between November 2020 and May 2021, with students completing follow-up surveys at 6 and 12 months after baseline data collection (final follow-up data were collected by May 2022). All students (intervention and control conditions) who provided consent and had parental consent were asked to complete the web-based self-report surveys in a supervised classroom setting. Students were assigned a unique participant identifier generated in REDCap (Research Electronic Data Capture; Vanderbilt University), a secure web-based data collection system. This identifier was used to email students personalized survey links. The unique code linked student data over time while maintaining confidentiality. Absent students were contacted by the research team (using details provided upon registration) and asked to complete the survey at home in their own time. Students who had left participating schools were contacted using secondary email addresses provided at baseline. Students were reimbursed for their participation with entry into a draw for 1 of 5 A$50 (US $34) Prezzee gift vouchers at each survey occasion.

### Measures

#### Mental Health and Substance Use Knowledge (Literacy)

Mental health and substance use knowledge (literacy) was measured by summing responses from a 12-item knowledge scale developed to measure topics covered by the program. Participants responded true or false to items such as “the Australian guidelines say that for people under the age of 18, the safest option is to not drink alcohol at all” and “a pounding heart, feeling sick and sweating can all be symptoms of anxiety.” Higher scores reflect greater mental health and substance use literacy.

#### Mental Health Measures

Anxiety symptoms in the past 2 weeks were assessed using the Generalized Anxiety Disorder 7-item scale [20], whereas depression symptoms over the past 2 weeks were assessed using the Patient Health Questionnaire Adolescent version [21]. General psychological distress in the past month was measured using the Kessler 6 scale [22]. Higher scores on these measures reflect higher levels of generalized anxiety, depression, and psychological symptoms.

#### Alcohol and Other Substance Use

The frequency of alcohol use was assessed using an adapted version of the Patterns of Alcohol Index [23]. Participants were asked, “How often did you have a standard alcoholic drink of any kind in the past 6 months?” (full drink). To aid in accurate reporting, a standard drinks chart was presented with these items. Responses were dichotomized to indicate whether participants had consumed at least 1 standard alcoholic drink in the preceding 6 months (0=no; 1=yes).

Other drug use was assessed using items from the Australian Institute of Health and Welfare: National Drug Strategy Household Survey [24]. Students were asked to report “Yes I have tried” or “No I have never tried” in relation to the following drugs: tobacco, vaping, cannabis, ecstasy, cocaine, methamphetamines, nonmedical use of prescription drugs, or a combination of drugs at the same time. A composite dichotomous score was calculated across all drug types (0=have never tried drugs; 1=have tried at least 1 drug).

#### Help-Seeking Measures

An item from the General Help-Seeking Questionnaire assessed future help-seeking intentions from a friend [25]. Using a 7-point scale, students rated the likelihood of seeking support from a friend for an emotional problem. To aid interpretability, this item was dichotomized (0=response options ranging from “extremely unlikely” to “unlikely”; 1=response options ranging from “likely” to “extremely likely”). Participants were also asked to indicate whether they sought help from a friend (not related to them) in the past 2 weeks (0=no; 1=yes). This item was obtained from the Actual Help-Seeking Questionnaire [26].

### Statistical Analysis

As per our preregistered analysis plan, multilevel mixed effect regression models were used to determine the effects of the intervention in Stata (version 17.0; StataCorp) [27,28]. The mixed effect repeated measures approach examines change over time both within and between groups while accounting for baseline differences and attrition. The intervention effects over time are modeled by a group-by-time interaction, where time is coded as a continuous linear variable representing baseline and the 6-month and 12-month follow-up assessments. The intraclass correlation showed that less than 10% of the variance existed at the school level; thus, 3-level models were abandoned instead of 2-level models for all outcomes [29]. All models included a random intercept at the individual level to account for the correlation between repeat measures over time, and a random slope was added when it improved the model fit, and the best-fitting covariance structure was tested using likelihood ratio tests, including the Akaike Information Criterion [30]. Time was coded as a continuous linear variable representing baseline and the 6-month and 12-month follow-up assessments. The time main effect represents the estimated 1-year change in the outcome in the control group over time, and the group main effect indicates differences in dependent variables between the intervention group and the control at baseline (referent category was the control group). To address the primary aims of the trial, intervention efficacy is modeled using a group-by-time interaction, representing the relative change in odds (logistic outcomes: alcohol use, drug use, help-seeking intentions, and help-seeking behavior) or mean frequency (continuous outcomes: knowledge [literacy], depression, psychological distress, generalized anxiety, and help seeking) in each outcome over 12 months. The interaction term for the binary outcomes was modeled using the *logit link function*, and the odds were calculated by exponentiating the regression coefficient. All continuous dependent outcomes were modeled using multilevel effect analysis. The assumption of normality was examined and sensitivity analyses with transformed data were conducted when the normality assumptions were violated. Full information maximum likelihood procedures were used to handle missing data, whereby all available data were included in the model estimates under the assumption that data were missing at random, and analyses were conducted on the intention-to-treat sample.

## Results

### Baseline Characteristics

**Figure 1:**
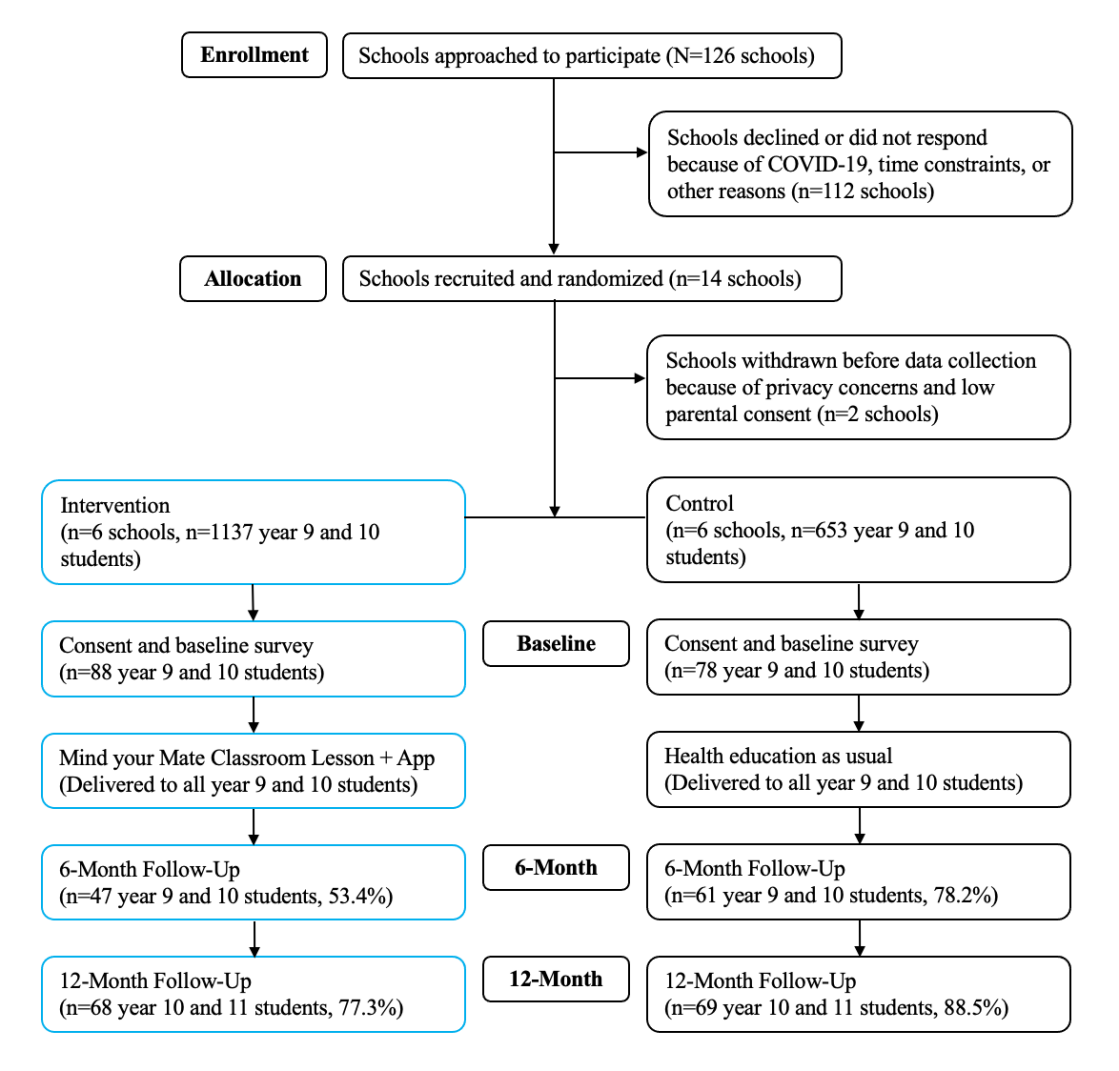
Flowchart of the recruitment, randomization, and assessment of participants.

A total of 166 students (mean age 15.3, SD 0.41 years; 72/166, 43.4% female; and 133/166, 80.1% born in Australia) completed the baseline assessment. Table 1 shows the baseline demographic characteristics by trial group, and Table 2 shows the frequency of each outcome over time. The recruitment details and follow-up rates are illustrated in the CONSORT (Consolidated Standards of Reporting Trials) diagram (Figure 1).

**Table 1:** Sample characteristics at baseline (N=166).

Characteristic			Control group (n=78)		MYM^a^ intervention group (n=88)
Age (years), mean (SD)			15.2 (0.4)		15.2 (0.4)
**Gender, n (%)**					
	Male	45 (58)		38 (43)	
	Female	28 (36)		44 (50)	
	Prefer not to say	2 (3)		3 (3)	
	Non-binary	3 (4)		3 (3)	
**Average grades (%), n (%)**					
	90-100	19 (24)		16 (18)	
	80-89	27 (35)		39 (44)	
	70-79	20 (26)		21 (24)	
	60-69	7 (9)		7 (8)	
	50-59	4 (5)		4 (5)	
	≤49	1 (1)		1 (1)	
**Country of birth, n (%)**					
	Australia	67 (86)		66 (75)	
	Other country	11 (14)		22 (25)	

^a^MYM: Mind your Mate.

**Table 2:** Outcome measures by trial group over time.

Outcome and time point		Control group	MYM^a^ intervention group
**Total knowledge score, mean (SD)**			
	Baseline	8.06 (2.12)	8.57 (1.54)
	0.5 y	8.70 (1.41)	8.83 (1.42)
	1 y	8.87 (2.01)	8.99 (1.32)
**Generalized anxiety score, mean (SD)**			
	Baseline	6.14 (5.31)	8.06 (5.66)
	0.5 y	6.33 (5.86)	7.73 (5.55)
	1 y	6.57 (6.11)	7.48 (5.68)
**Depressive symptoms, mean (SD)**			
	Baseline	6.47 (5.10)	9.33 (6.59)
	0.5 y	7.21 (6.18)	8.82 (5.64)
	1 y	7.61 (5.93)	8.33 (6.58)
**Psychological distress, mean (SD)**			
	Baseline	9.28 (5.57)	10.31 (5.69)
	0.5 y	8.70 (5.80)	10.18 (5.41)
	1 y	9.52 (6.15)	10.70 (6.10)
**Full alcoholic drink, n/N (%)**			
	Baseline	29/76 (38)	9/86 (10)
	0.5 y	13/59 (22)	9/46 (20)
	1 y	29/69 (42)	17/67 (25)
**Substance use, n/N (%)**			
	Baseline	28/75 (37)	14/86 (16)
	0.5 y	18/57 (32)	10/45 (22)
	1 y	29/68 (43)	16/67 (24)
**Intentions to help a friend, n/N (%)**			
	Baseline	48/70 (69)	49/78 (63)
	0.5 y	39/55 (71)	34/43 (79)
	1 y	40/61 (66)	39/60 (65)
**Actual help to a friend, n/N (%)**			
	Baseline	22/72 (31)	39/84 (46)
	0.5 y	26/57 (46)	22/44 (50)
	1 y	25/65 (38)	34/66 (52)

^a^MYM: *Mind your Mate*.

### Outcome of Health and Education Measures

Coefficients from the mixed effects regression models for all outcomes are presented in Table 3 and 4. The estimated marginal means and probability plots for each outcome can be found in Figures S1 to S8 in Multimedia Appendix 1.

**Table 3:** Relative annual change in the growth of study main outcomes over 12 months for students allocated to the Mind your Mate intervention compared with that of the control group.

Outcome		b (95% CI)	*P* value	Effect size (Cohen *d*)
**Total knowledge (literacy) score**				
	Group main effect^a^	0.43 (−0.07 to 0.94)	.10	0.25
	Time main effect^b^	*0.77* ^c^ *(0.40 to 1.13)*	*<.001*	0.45
	Group × time interaction^d^	−0.37 (−0.88 to 0.14)	.15	−0.22
**Depression scores**				
	Group main effect	*2.76 (1.01 to 4.51)*	*.002*	0.46
	Time main effect	1.18 (−0.16 to 2.52)	.08	0.20
	Group × time interaction	−*1.86 (−3.73 to 0.02)*	*.05*	−0.31
**Psychological distress scores**				
	Group main effect	0.97 (−0.69 to 2.63)	.25	0.17
	Time main effect	0.17 (−1.22 to 1.56)	.81	0.03
	Group × time interaction	0.38 (−1.56 to 2.31)	.70	0.07
**Generalized anxiety scores**				
	Group main effect	*1.84 (0.11 to 3.57)*	*.03*	0.32
	Time main effect	0.47 (−0.86 to 1.79)	.48	0.08
	Group × time interaction	−0.77 (−2.61 to 1.08)	.41	−0.13

^a^Group main effect: group differences at baseline (referent group: control).

^b^Time main effect: change over time in the control group.

^c^Italicization indicates significance at *P*≤.05.

^d^Group × time interaction: change over time by group.

**Table 4:** Relative annual change in the growth of other study outcomes over 12 months for students allocated to the Mind your Mate intervention compared with that of the control group.

Outcome		OR^a^ (95% CI)	*P* value
**Alcohol use in the past 6 months (full drink)**			
	Group main effect^b^	*0.01 (0.00*-*0.22)*^c^	*<.01*
	Time main effect^d^	1.94 (0.42-9.05)	.40
	Group × time interaction^e^	8.95 (0.69-116.49)	.09
**Other substance use**			
	Group main effect	*0.06 (0.01*-*0.49)*	*<.01*
	Time main effect	2.36 (0.66-8.46)	.19
	Group × time interaction	1.51 (0.22-10.26)	.67
**Intentions to seek help from friend**			
	Group main effect	0.65 (0.26-1.63)	.36
	Time main effect	0.74 (0.31-1.78)	.51
	Group × time interaction	1.45 (0.44-4.75)	.54
**Actual help seeking from friend**			
	Group main effect	2.42 (0.89-6.58)	.09
	Time main effect	2.03 (0.81-5.04)	.13
	Group × time interaction	0.66 (0.19-2.25)	.50

^a^OR: odds ratio.

^b^Group main effect: group differences at baseline (referent group: control).

^c^Italicization indicates significance at *P*≤.05.

^d^Time main effect: change over time in the control group.

^e^Group × time interaction: change over time by group.

#### Mental Health and Substance Use Knowledge (Literacy)

At baseline, there was no difference in the average knowledge (literacy) scores between the trial groups (b=0.43, 95% CI −0.07 to 0.94; Cohen *d*=0.25). Although the average knowledge score increased moderately in the control group over time (b=0.77, 95% CI 0.40-1.13; Cohen *d=*0.45), there was no difference in the rate of change in scores between the groups over the 12-month follow-up period (b=−0.37, 95% CI −0.88 to 0.14; Cohen *d*=−0.22).

#### Mental Health Measures

For depressive symptoms, there was a moderate-sized group main effect, indicating that the intervention group had higher depression scores than the control group at baseline (b=2.76, 95% CI 1.01-4.51; Cohen *d=*0.46). There was no substantial change in depressive symptoms in the control group over time (b=1.18, 95% CI −0.16 to 2.52; Cohen *d=*0.20). The group-by-time interaction indicates that students in the intervention group experienced reductions in depressive symptoms over the 12-month period compared with students in the control group and this was a small to moderate effect (b=−1.86, 95% CI −3.73 to 0.02; Cohen *d=*−0.31). Psychological distress scores did not differ between groups at baseline (b=0.97, 95% CI −0.69 to 2.63; Cohen *d=*0.17), did not show change over time in the control group (b=0.17, 95% CI −1.22 to 1.56; and Cohen *d=*0.03), and there was no difference in the rate of growth over time between the trial groups (b=0.38, 95% CI −1.56 to 2.31; Cohen *d=*0.07). There was a small-sized group main effect showing that generalized anxiety scores were higher at baseline in the intervention group than in the control group (b=1.84, 95% CI 0.11 to 3.57; Cohen *d=*0.32). However, the control group did not show changes over time (b=0.47, 95% CI −0.86 to 1.79; Cohen *d=*0.08), and there was no difference in the rate of growth over time between the groups (b=−0.77, 95% CI −2.61 to 1.08; Cohen *d=−*0.13).

Despite depressive symptoms, psychological distress, and generalized anxiety following a near-normal distribution, a sensitivity analysis was conducted to examine any impact of nonnormality. The best normality transformations were identified, and the analyses were rerun using the transformed data (Multimedia Appendix 1). In all cases, there was no substantive difference in results using transformed data, and thus, results from raw data models are reported for ease of interpretation.

#### Alcohol and Other Substance Use

There was a very small–sized group main effect, showing that alcohol use in the past 6 months was higher in the intervention group than in the control group at baseline (odds ratio [OR] 0.01, 95% CI 0.01-0.22). Alcohol use did not vary over time in the control group (OR 1.94, 95% CI 0.42- 9.05), and there was no evidence of group differences over time (OR 8.95, 95% CI 0.69-116.49), although large variability was observed in this estimate, therefore increasing the uncertainty around the OR observed. Similarly, for other substance use, there was a very small main effect for group at baseline (OR 0.06, 95% CI 0.01-0.49), although there was no evidence of a change in the odds over time in the control group (OR 2.36, 95% CI 0.66-8.46) or a difference between the groups over the 12-month follow-up (OR 1.51, 95% CI 0.22-10.26).

#### Help-Seeking Measures

Intentions to seek help from a friend did not change over time in the control group (OR 0.74, 95% CI 0.31-1.78), and there was no evidence of a between-group difference at baseline (OR 0.65, 95% CI 0.26-1.63). Over the 12-month follow-up, there was no evidence of a group difference in intentions to seek help (OR 1.45, 95% CI 0.44-4.75). Similarly, actual help seeking from a friend was not significantly different between groups at baseline (OR 2.42, 95% CI 0.89-6.58), did not change over time in the control group (OR 2.03, 95% CI 0.81-5.04), or was not different between groups over time (OR 0.66, 95% CI 0.19-2.25).

### Uptake and Adherence

Of the 88 students in the *Mind your Mate* condition, 43 (49%) downloaded and registered an account on the mobile app. These students accessed the app between 1 and 7 times, opening it an average of 1.7 (SD 1.6) times. A mean of 5.2 (SD 12.6) days passed between the first and last time the students opened the app. The maximum number of days between the first and last time a student used the app was 48 days.

**Figure 2:**
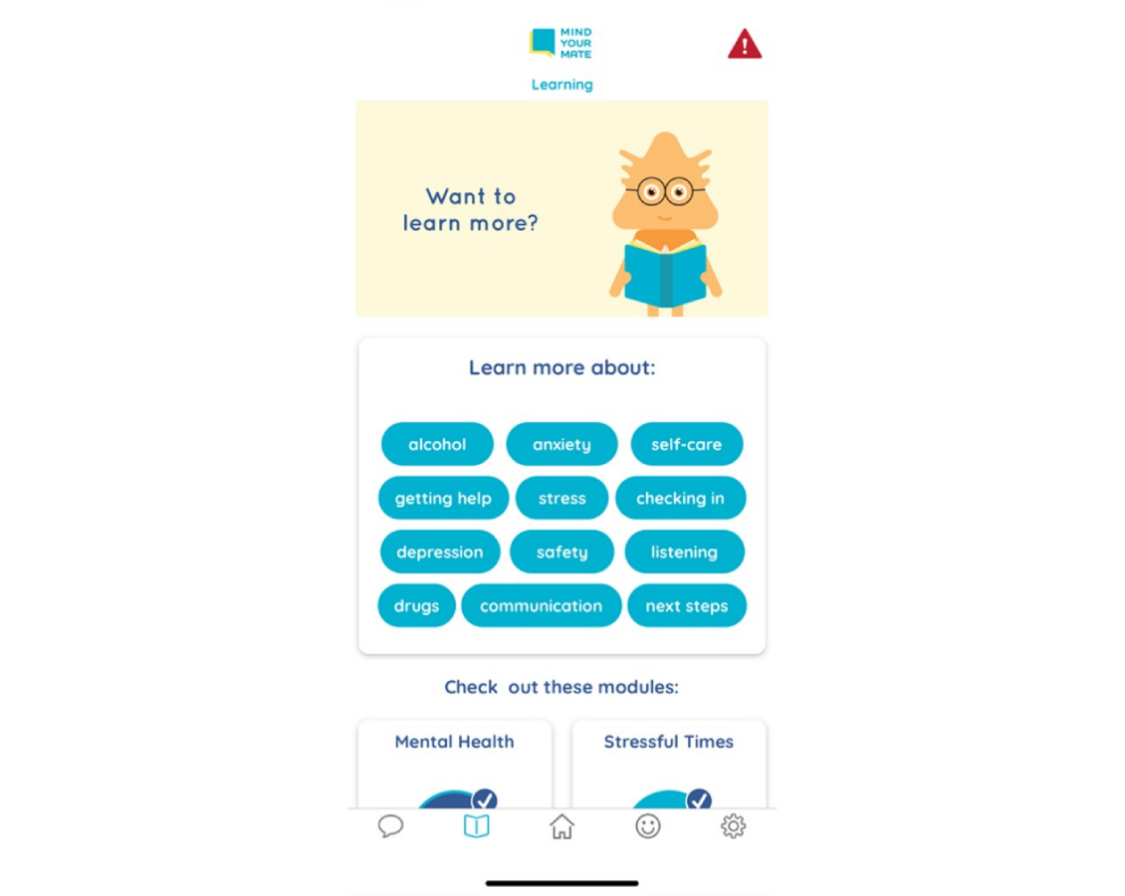
Screenshot of the Mind your Mate app.

Data were collected on the use of key functions in the app. The educational modules were the most popular function accessed, seen in Figure 2. Of the registered users, 33% (14/43) opened at least 1 and up to 4 different educational modules. The mean number of modules opened by a student was 1.57 (SD 0.9) modules, with 5 students opening ≥1 module. The mood tracker function was used by 23% (10/43) of registered users. These students logged at least 1 mood entry, with an average of 1.3 (SD 0.5) mood entries. In total, 12 entries were made in the mood tracker. The friend function was used by 8 students to create 9 friend profiles. Of the registered users, 19% (8/43) created a profile about a friend, with 1 student making 2 friend profiles. The conversation-planning function and the self-care activity function were not used by any student in the trial.

### Acceptability

Acceptability analysis measured a subset of intervention participants who met the following criteria: (1) those who completed the app evaluation section of the surveys and (2) those who indicated that they downloaded the app. A total of 25 participants at the 6-month follow-up and 32 participants at the 12-month follow-up met the criteria for this subset. The open-ended responses were thematically analyzed. At the 6-month follow-up, 36% (9/25) of students had used the app, and 20% (5/25) reported using it once or twice; at the 12-month follow-up, this increased to 50% (16/32) of students having used the app and 41% (13/32) having used it once or twice.

Students who at least “slightly agreed” that they liked the *Mind your Mate* app accounted for 48% (12/25) and 44% (11/25) of this subset at 6-month and 12-month follow-ups, respectively. Throughout the intervention, the average 5-star rating of the app remained stable from 3.48 to 3.38 stars (SD 0.57 and 0.82 at 6-month and 12-month follow-ups, respectively). Most students indicated that “maybe” there are “several people” to whom they would recommend the app. When asked about the likelihood of the app increasing their mental health and well-being awareness, those who at least “slightly agreed” made up 56% (14/25) at the 6-month and 12-month follow-ups. Regarding the app’s usefulness, over a third of the students agreed that it was useful (6 months: 9/25, 36% and 11/32, 34%) at both follow-up occasions. At the 6-month and 12-month follow-ups, about half of the students' responses varied from “slightly agree” to “strongly agree” to indicate the app is likely to encourage further help seeking (12/25, 48% and 15/32, 47%).

In a separate subset of students who did not download the app, open-ended responses regarding why they did not download the app were thematically analyzed. A total of 15 responses were captured at the 6-month follow-up and 20 responses at the 12-month follow-up. Participants’ reasons for not downloading the app were as follows, from most to least frequent: no motivation (n=3 and 5 at 6-month and 12-month follow-ups, respectively), not useful (n=2 and 6 at 6-month and 12-month follow-ups, respectively), forgot to download it (n=2 and 3 at 6-month and 12-month follow-ups, respectively), high storage requirement (n=2 and 3 at 6-month and 12-month follow-ups, respectively), missed the lesson with download instructions (n=2 and 2 at 6-month and 12-month follow-ups, respectively), no time to use the app (n=1 and 1 at 6-month and 12-month follow-ups, respectively), no one to connect to (n=1 and 1 at 6-month and 12-month follow-ups, respectively), and privacy concerns (n=1 and 0 at 6-month and 12-month follow-ups, respectively). The full details of the responses can be found in Multimedia Appendix 1.

## Discussion

### Principal Findings

This is the first trial to test a novel digital health peer intervention, *Mind your Mate,* for the prevention of mental health problem and substance use problem in adolescents. The program aimed to foster peer support among adolescents using digital technology. *Mind your Mate* was found to result in reductions in depressive symptoms among students in the intervention group up to 12 months after baseline when compared with a control condition, and the size of this effect was small to moderate. No differences were observed between the intervention and control group in relation to all other outcomes (ie, mental health and substance use literacy, anxiety symptoms, substance use, and help seeking).

The impact on depression symptoms is notable (Cohen *d=*−0.31), given that school-based universal programs for depression typically show small to nil effects [31]. Targeted programs commonly show larger effects than universal programs for depression symptoms [32,33]; however, most of these programs involve multiple classroom sessions, often facilitated by external providers, and require significant training and resources [31,32]. The current digital intervention was less intensive, consisting of 1 web-based lesson and mobile app, providing the potential to deliver the intervention widely at a relatively low cost, with no additional training required from the teachers or schools. *Mind your Mate* also takes a transdiagnostic approach covering multiple mental health and substance use concerns. Although depression content is covered, it is not the sole focus of the program, which instead aims to foster peer support. The principle of peer support embedded within the intervention aims to strengthen and foster adolescents’ social connections, both within their peer groups and with trusted adults. Social connection is known to be a key protective factor for mental health problems. In this context, the impact on depression symptoms is notable. Anxiety symptoms decreased among students in the intervention group; however, these reductions did not meet the statistical significance thresholds, and it would be worthy of further exploration in a larger controlled effectiveness trial.

The program’s lack of impact on knowledge (literacy) and help-seeking contrasts with other universal mental health prevention programs, which typically find effects on knowledge, with help seeking, symptom, and stigma reduction being harder to demonstrate [34-36]. In this study, baseline knowledge scores were relatively high and remained stable across the study in both groups, indicating that the current sample of adolescents had good mental health and substance use literacy in being able to accurately identify symptoms of common mental disorders and related risk factors. For example, 85.4% (135/158) of the students agreed or strongly agreed that “good sleep helps improve mental health” when asked at baseline. This may reflect the increasing prominence of mental health campaigns in the public domain, including across social media [37], as well as the increasing uptake of well-being and mental health literacy programs in schools [34]. However, despite high literacy levels, help-seeking behavior was low, and a substantial proportion of students experienced poor mental health symptoms over the course of the study in both groups. This indicates that future prevention efforts may be better served by focusing on active strategies and tools that go beyond literacy and proactively prevent mental health and substance use problems, including early help seeking. It is also noteworthy that the trial was conducted during the initial years of the COVID-19 pandemic from November 2020 to May 2022, a time of unprecedented change and stress for adolescents who were transitioning in and out of home schooling and were facing limited interaction with peers. It is possible that these environmental stressors outweighed this intervention’s preventive capacity and that a higher-intensity intervention was needed during these years to support mental health. Nevertheless, promising findings were demonstrated in relation to depression symptoms, with other outcomes in the hypothesized directions.

Another possibility for a lack of significant findings was an overall lack of engagement with the mobile app component of the intervention. Approximately half of the students in the intervention condition registered an account on the mobile app, and among these students, regular use of the app was low. This is not uncommon in mobile health intervention studies. For example, Werner-Seidler et al [38] found that 47% of their participants aged 12 to 16 years in an insomnia mobile cognitive behavioral therapy intervention did not use the app because there was too much text to read. Several strategies were used to enhance engagement with the mobile app. In addition to the classroom lesson prompting students to download the app, teachers were instructed to remind students to download and engage with the app, and an email was sent to all students at the 6-month follow-up survey. Two pop-up notification reminders were also sent to those who downloaded the app in a 2.5-week period around the 6-month follow-up. However, student responses indicated that some students were not clearly instructed to download and engage with the app. This points to clearer implementation guides or, potentially, the use of outside facilitators to guide the introductory lesson. It is also possible that the demands of COVID-19 may have impacted the teacher’s abilities to implement and follow-up on the student app use among additional workloads at the time.

Among those students who did use the app, acceptability of the intervention was high. Nearly half of the intervention participants downloaded and registered an account on the app (43/88, 49%), and at the 12-month app evaluation, many at least “slightly agreed” that they liked the *Mind your Mate* app (14/32, 44%). However, a lack of overall adherence shows that there is room for improvement, such as students requesting a “more in-depth ‘friends’ system.” This is in line with a systematic review of engagement of children and young people in digital mental health interventions that found higher than average adherence among digital interventions that included the following features: videos, limited text, ability to personalize, ability to connect with others, and options to receive reminder SMS text messages [39]. Future research using the app may also benefit from the inclusion of further qualitative data collection to better understand how the app made an impact, including interviews with the adolescents and possibly their parents.

### Strengths and Limitations

Key strengths of this study include the use of a rigorous randomized controlled design with preregistration of outcomes and hypotheses [18] and the inclusion of students from both public and private schools. Excellent follow-up rates were achieved with 83% follow-up at 12 months, exceeding the average retention rates for digital health intervention studies among children and young people [39]. Despite these strengths, our findings must be considered in light of several limitations. Although we were able to recruit our target number of schools, student participation within the schools was lower than anticipated, largely because of a lack of return of active parental consent forms. Parental consent was heavily impacted by the COVID-19 pandemic, which placed extreme pressure on school staff and parents during the time in which consent forms were distributed for this study. Although the research team worked closely with schools to implement a range of strategies including multiple forms of consent (eg, electronic and hard copy), reminder emails, and articles in school newsletters, the resulting consent rate was reflective of the challenging time in which consent for the research study was conducted. Importantly, although consent was required to participate in the surveys, all students at the participating schools received the intervention component as it mapped onto their existing curriculum. The research team also worked closely with schools to be flexible at the start of the study, as recruitment took longer than anticipated owing to disruptions in school closures because of the COVID-19 pandemic. It is acknowledged that analyses may be underpowered to detect significant effects on all outcomes, and replication in a larger-scale RCT is needed to confirm the efficacy of the program.

### Conclusions

In conclusion, the novel digital health universal peer intervention, *Mind your Mate*, resulted in reduced depression symptoms over 12 months in adolescents compared with those in a control condition. There is a need to continue to refine, develop, and evaluate innovative applied approaches to the prevention of mental disorders in real-world settings.

## Appendix

Multimedia Appendix 1 Sensitivity analyses, additional model outputs, acceptability and evaluation data.

Multimedia Appendix 2 CONSORT-eHEALTH checklist (V 1.6.1).

## Abbreviations

CONSORT: Consolidated Standards of Reporting Trials
NSW: New South Wales
OR: odds ratio
RCT: randomized controlled trial
REDCap: Research Electronic Data Capture

## Data Availability

The data collected for the study, including individual participant data, will be made available to researchers upon request to the study team and with appropriate reason, when accompanied by the study protocol and analysis plan, and on approval by a committee of this research team. The shared data will be deidentified participant data. Data will be shared after approval of a proposal with a signed data access agreement. Proposals should be directed to the corresponding author.
