# Supplementary material for: Evaluating a Peer-Support Mobile App for Mental Health and Substance Use Among Adolescents Over 12 Months During the COVID-19 Pandemic: Randomized Controlled Trial
Source: J Med Internet Res. 2023 Sep 27;25:e45216. doi: 10.2196/45216 (PMC10538359; doi:10.2196/45216)
Supplement: Multimedia Appendix 1 [file jmir_v25i1e45216_app1.docx]

|  | ***b*** | **95% CI** | **p-value** |
| --- | --- | --- | --- |
| **Depression scores** |  |  |  |
| Time main effect | 0.17 | [-0.05 – 0.38] | 0.12 |
| Group main effect | **0.48** | **[0.20 – 0.76]** | **<0.01** |
| Group x time interaction | **-0.34** | **[-0.64 - -0.04]** | **0.03** |
| **Psychological distress scores** |  |  |  |
| Time main effect | <0.01 | [-1.24 – 0.24] | 0.99 |
| Group main effect | 0.18 | [-0.10 – 0.45] | 0.22 |
| Group x time interaction | 0.07 | [-0.26 – 0.40] | 0.69 |
| **Generalised anxiety scores** |  |  |  |
| Time main effect | 0.04 | [-0.16 – 0.25] | 0.68 |
| Group main effect | **0.32** | **[0.04 – 0.60]** | **0.03** |
| Group x time interaction | -0.12 | [-0.39 – 0.18] | 0.47 |

**Supplementary material**

**Table S1.** Relative annual change in the growth of normality transformed outcomes over 12 months for students allocated to the *MindYourMate* intervention compared to the control group

**Figure S1.** Model-estimated mean knowledge (literacy) score by group across all time points.


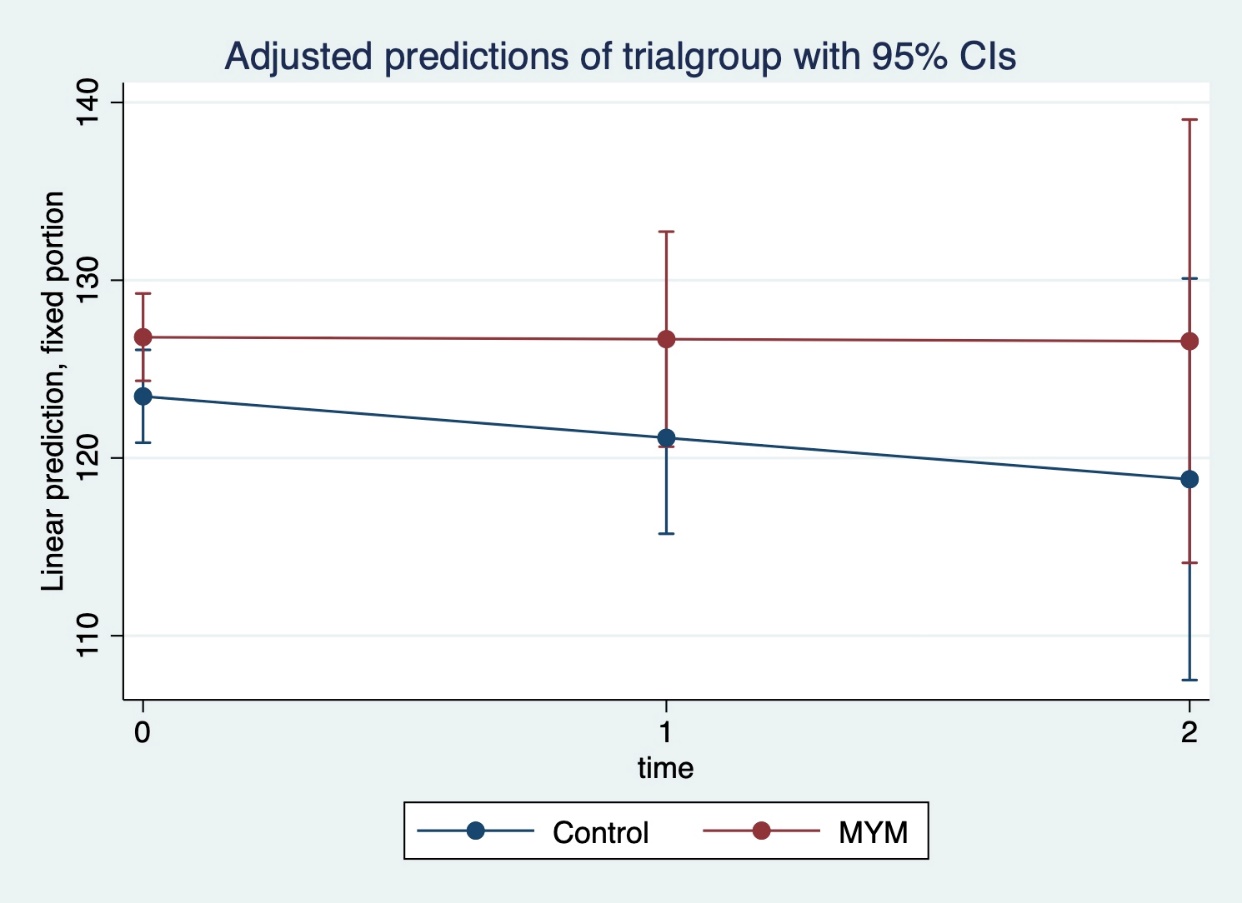


**Figure S2.** Model-estimated probability of consuming a full serve of alcohol in the past 6-months by group across all time points.


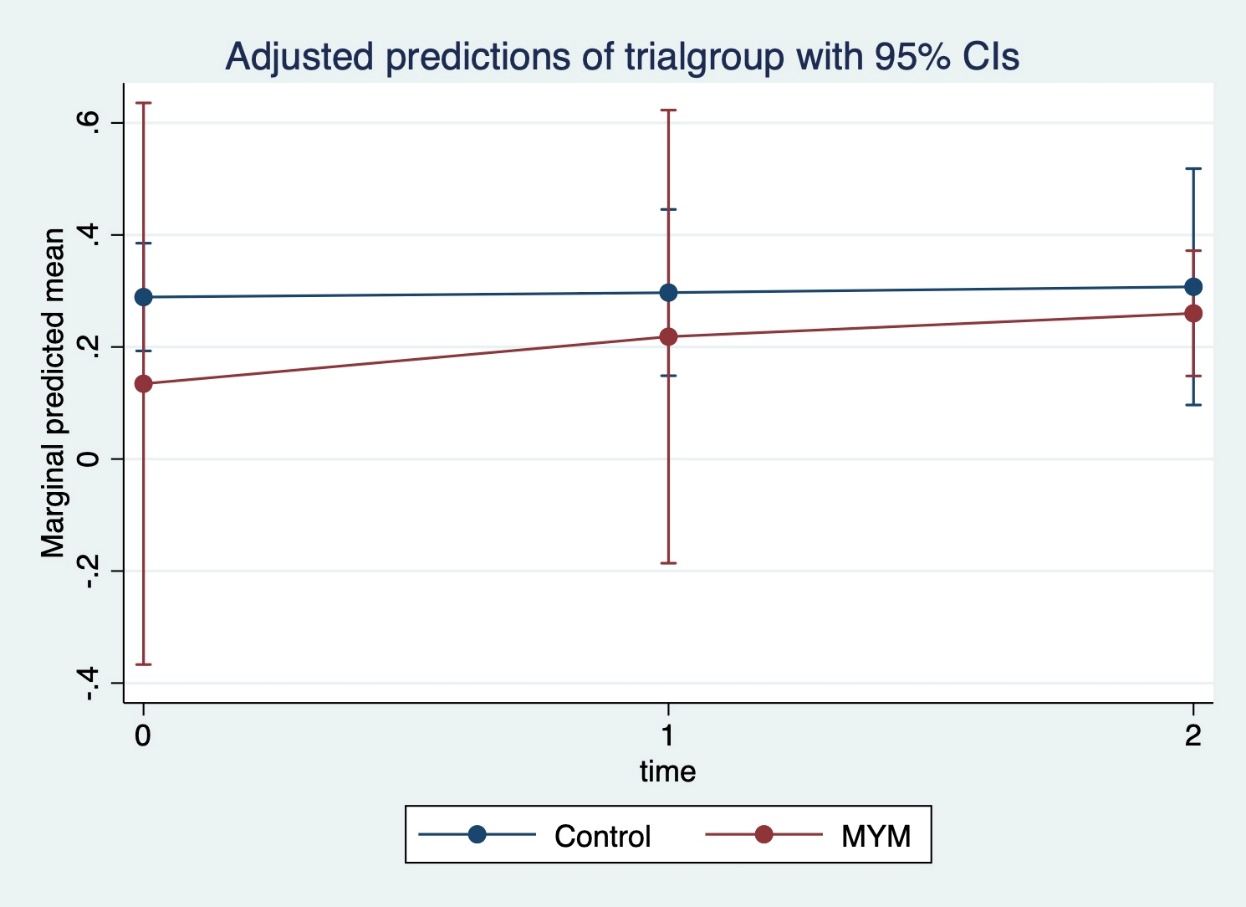


**Figure S4.** Model-estimated mean GAD-7 anxiety score by group across all time points.


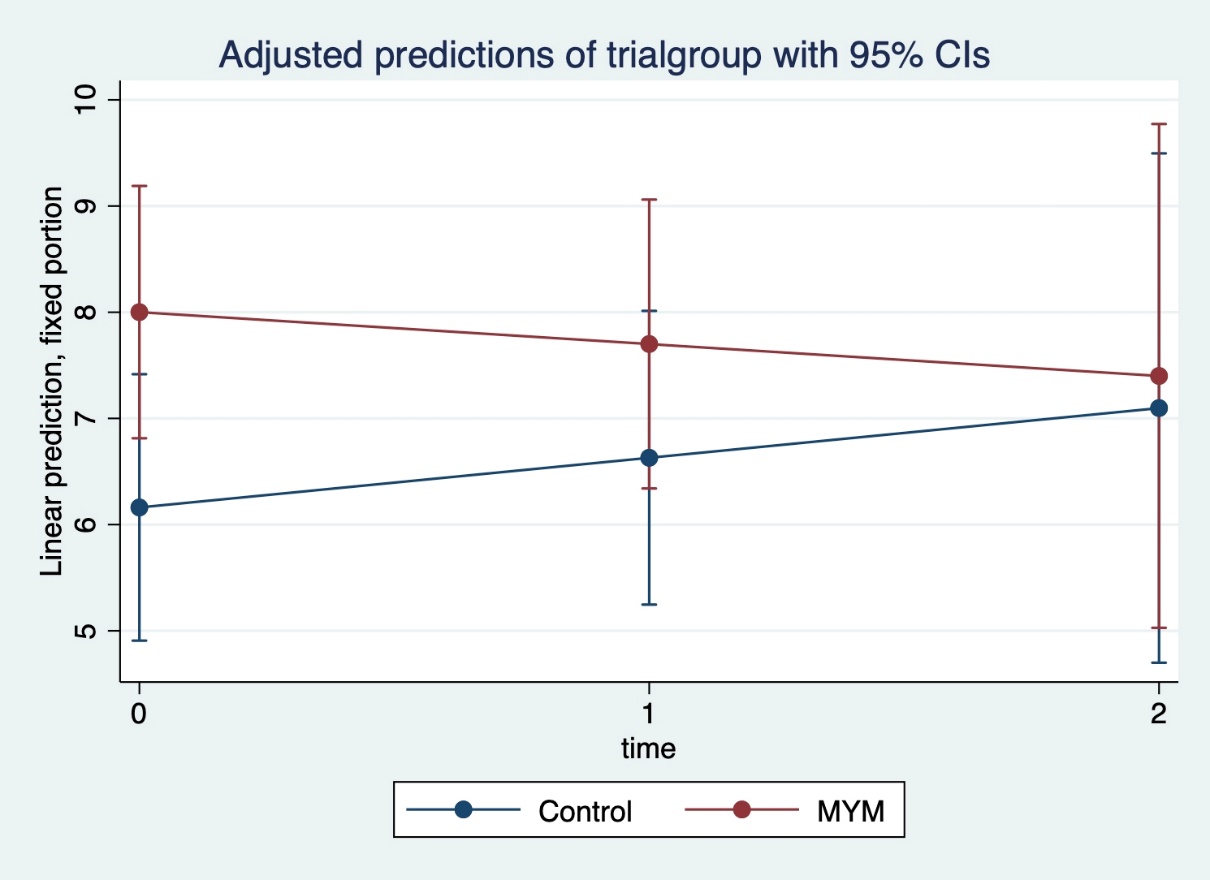


**Figure S5.** Model-estimated mean PHQ-A depression score by group across all time points.


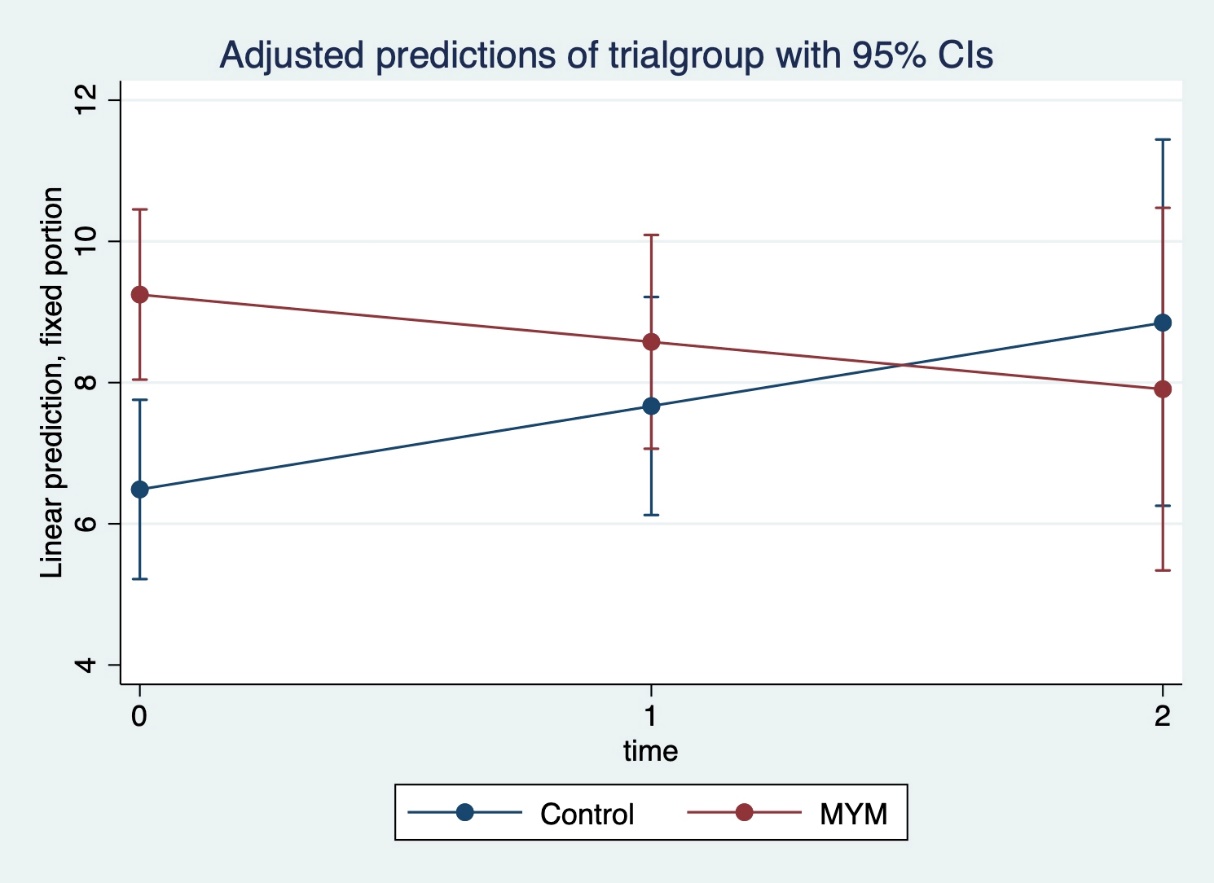


**Figure S5.** Model-estimated mean psychological distress (K6) score by group across all time points.


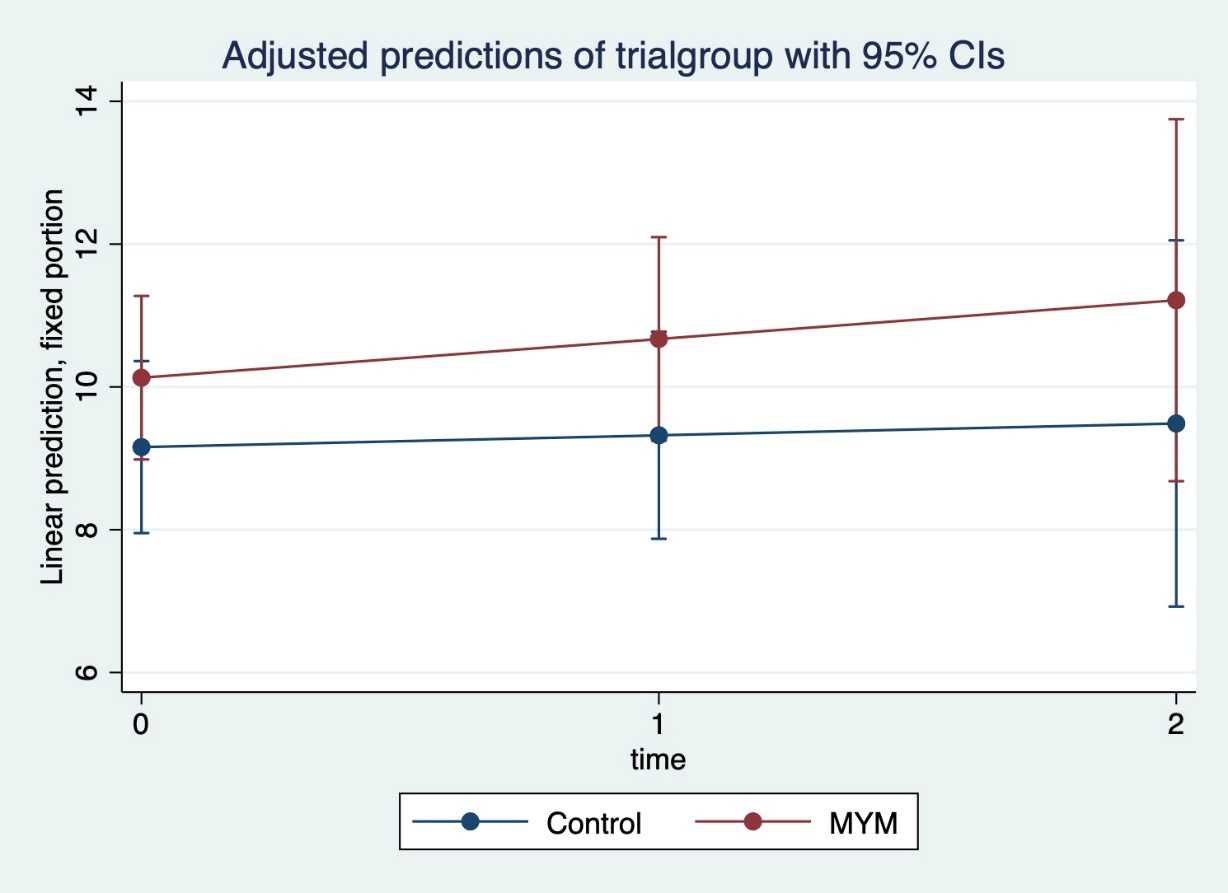


**Figure S6.** Model-estimated probability of intentions to seek help from a friend by group across all time points.


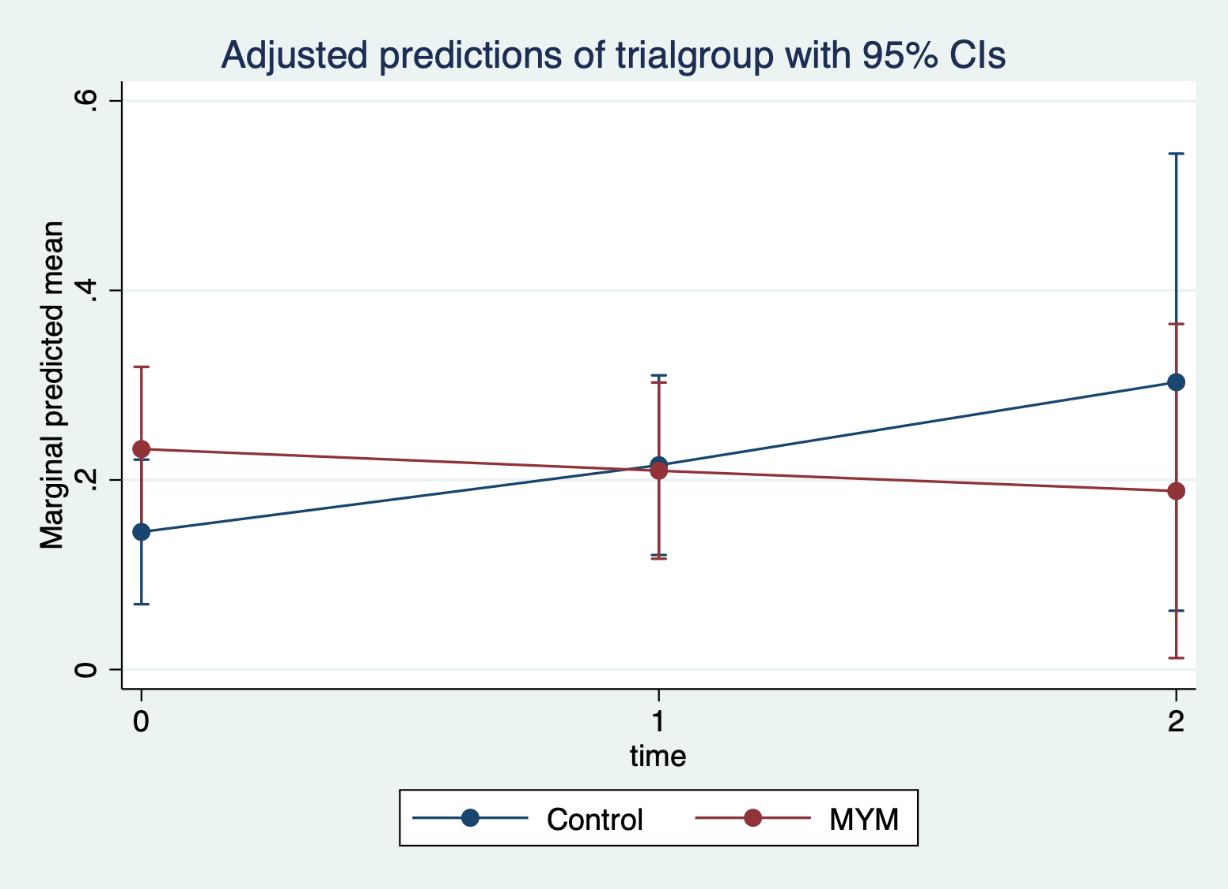


**Figure S7.** Model-estimated probability of actual help seeking from a friend by group across all time points.

**
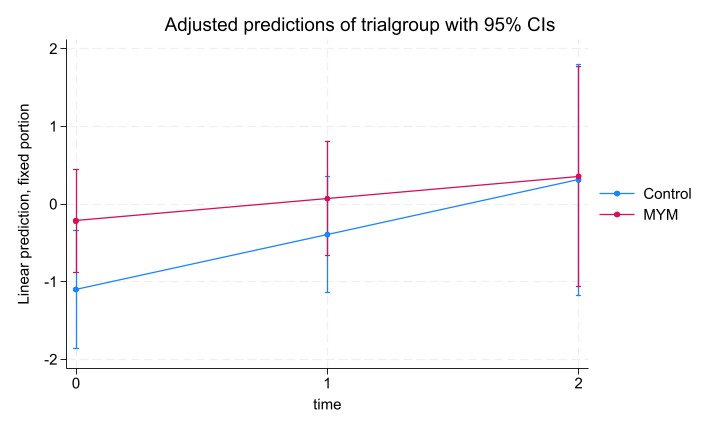
**

**Figure S8.** Model-estimated probability of substance use (other than alcohol) in the past 6-months by group across all time points.


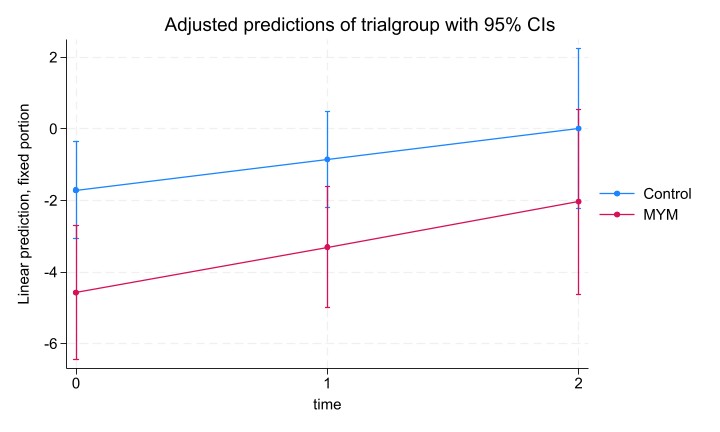


**Detailed acceptability analysis**

This acceptability analysis measured a subset of intervention participants who met the following criteria: (1) completed the app evaluation section of the surveys and (2) indicated that they downloaded the app. The total participants of this subset at the 6-month follow up is n=25; at the 12-month follow up n=32 participants met criteria of this subset.

| **Question** | **Survey instance** | **Response options and respective proportion of answers** | | | | | | |
| --- | --- | --- | --- | --- | --- | --- | --- | --- |
|  |  | **0 times** | **1-2 times** | **3-5 times** | **5-10 times** | **>10 times** |  |  |
| How many times did you use the ‘Mind your Mate’ app in the past 6 months? | T2 | 64% (n=16) | 20% (n=5) | 4% (n=4) | 0% | 0% |  |  |
|  | T3 | 50% (n=16) | 40.63% (n=13) | 9.38% (n=3) | 0% | 0% |  |  |
|  |  | **Strongly agree** | **Moderately agree** | **Slightly agree** | **Undecided** | **Slightly disagree** | **Moderately disagree** | **Strongly disagree** |
| Overall I liked the ‘Mind your Mate’ app | T2 | 4% (n=1) | 20% (n=5) | 24% (n=6) | 40% (n=10) | 8% (n=2) | 4% (n=1) | 0% |
|  | T3 | 12.5% (n=4) | 9.38% (n=3) | 21.88% (n=7) | 46.88 (n=15) | 3.13% (n=1) | 3.13% (n=1) | 3.13% (n=1) |
| The ‘Mind your Mate’ app is useful | T2 | 0% | 24% (n=6) | 20% (n=5) | 44% (n=11) | 4% (n=1) | 4% (n=1) | 4% (n=1) |
|  | T3 | 9.38% (n=3) | 9.38% (n=3) | 15.63% (n=5) | 40.63% (n=13) | 18.75% (n=6) | 0% | 6.25% (n=2) |
| The videos in the app helped keep my interest while I learnt the information | T2 | 0% | 24% (n=6) | 12% (n=3) | 56% (n=14) | 4% (n=1) | 4% (n=1) | 0% |
|  | T3 | 12.5% (n=4) | 9.8% (n=3) | 9.8% (n=3) | 56.25% (n=18) | 6.25% (n=2) | 0% | 6.25% (n=2) |
| The information was relevant to current or future experiences in my life or lives of my peers | T2 | 4% (n=1) | 16% (n=4) | 24% (n=6) | 44% (n=11) | 8% (n=2) | 4% (n=1) | 0% |
|  | T3 | 9.38% (n=3) | 12.5% (n=4) | 15.63% (n=5) | 43.75% (n=14) | 6.25% (n=2) | 6.25% (n=2) | 6.25% (n=2) |
| The information was easy to understand | T2 | 16% (n=4) | 20% (n=5) | 16% (n=4) | 44% (n=11) | 4% (n=1) | 0% | 0% |
|  | T3 | 15.63% (n=5) | 21.88% (n=7) | 9.38% (n=3) | 46.88% (n=15) | 3.13% (n=1) | 0% | 3.13% (n=1) |
| The information was easy to learn | T2 | 8% (n=2) | 24% (n=6) | 16% (n=4) | 40% (n=10) | 8% (n=2) | 4% (n=1) | 0% |
|  | T3 | 15.63% (n=5) | 21.88% (n=7) | 15.63% (n=5) | 37.5% (n=12) | 6.25% (n=2) | 0% | 3.13% (n=1) |
| Use of this app is likely to encourage further help seeking (if it’s required) | T2 | 0% | 20% (n=5) | 28% (n=7) | 40% (n=10) | 8% (n=2) | 4% (n=1) | 0% |
|  | T3 | 15.63% (n=5) | 18.75% (n=6) | 12.5% (n=4) | 37.5% (n=12) | 9.38% (n=3) | 3.13% (n=1) | 3.13% (n=1) |
| I liked the ability to personalise the app | T2 | 8% (n=2) | 8% (n=2) | 20% (n=5) | 52% (n=13) | 8% (n=2) | 4% (n=1) | 0% |
|  | T3 | 25% (n=8) | 15.63% (n=5) | 6.25% (n=2) | 50% (n=16) | 0% | 0% | 3.13% (n=1) |
| I thought the app was age appropriate | T2 | 0% | 24% (n=6) | 28% (n=7) | 28% (n=7) | 16% (n=4) | 4% (n=1) | 0% |
|  | T3 | 25% (n=8) | 12.5% (n=4) | 9.38% (n=3) | 34.38% (n=11) | 6.25% (n=2) | 3.13% (n=1) | 9.38% (n=3) |
| This app is likely to increase awareness of mental health and wellbeing | T2 | 4% (n=1) | 36% (n=9) | 16% (n=4) | 32% (n=8) | 8% (n=2) | 4% (n=1) | 0% |
|  | T3 | 18.75% (n=6) | 21.88% (n=7) | 15.63% (n=5) | 28.13% (n=9) | 9.38% (n=3) | 3.13% (n=1) | 3.13% (n=1) |
| I plan to use the information I learnt in this app in my own life | T2 | 0% | 20% (n=5) | 16% (n=4) | 48% (n=12) | 8% (n=2) | 8% (n=2) | 0% |
|  | T3 | 15.63% (n=5) | 9.38% (n=3) | 18.75% (n=6) | 40.63% (n=13) | 3.13% (n=1) | 0% | 6.25% (n=2) |
|  |  | **Not at all. I would not recommend this app to anyone** | **There are very few people I would recommend this app to** | **Maybe. There are several people whom I would recommend it to** | **There are many people I would recommend this app to** | **Definitely. I would recommend this app to everyone** |  |  |
| Would you recommend this app to people who might benefit from it? | T2 | 12% (n=3) | 24% (n=6) | 36% (n=9) | 24% (n=6) | 4% (n=1) |  |  |
|  | T3 | 9.38% (n=3) | 25% (n=8) | 40.63% (n=13) | 15.63% (n=5) | 9.38% (n=3) |  |  |
|  |  | **One star, one of the worst apps I’ve used** | **Two stars** | **Three stars, average** | **Four stars** | **Five stars, one of the best apps I’ve used** |  |  |
| What is your overall star rating of the app? | T2 | 0% | 0% | 56% (n=14) | 40% (n=10) | 4% (n=1) |  |  |
|  | T3 | 3.13% (n=1) | 3.13% (n=1) | 56.25% (n=18) | 28.13% (n=9) | 9.38% (n=3) |  |  |
|  |  | **Yes** | **No** |  |  |  |  |  |
| Do you think it would be useful if the content and functions of the app were available even when you are not connected to the internet? | T2 | 92% (n=23) | 8% (n=2) |  |  |  |  |  |
|  | T3 | 93.75% (n=30) | 6.25% (n=2) |  |  |  |  |  |
| Are there any other features you would like to see in the app? | T2 | “A list of mental health professionals” (n=1)  “More videos” (n=1)  “more in depth ‘friends’ system” (n=1)  “Access to people without the school code” (n=1)  “I'd like to see a better user interface, the app was hard to navigate.” (n=1)  “Games” (n=1)  “Features of the app I would like to see. include abilities to reflect and communicate with similarly minded people.” (n=1) | | | | | | |
|  | T3 | “No wifi needed” (n=2)  “The information could be at a higher level, and more applicable to people supporting friends through a crisis.” (n=1)  “A list of mental health professionals in the local area and where to find financial support for it, if there is any” (n=1)  “The Mind your Mate App is a nice and helpful App.” (n=1)  “I'd like to see a better user interface, the app was hard to navigate.” (n=1)  “I think the app is good” (n=3) | | | | | | |
| Are there any other Are there any other comments you would like to make about the Mind your Mate app? you would like to see in the app? | T2 | “Useful” (n=2)  “Helpful” (n=1)  “I was unable to use the app as I didn't have the school code. Greater accessibility would be helpful.” (n=1) | | | | | | |
|  | T3 | “Useful” (n=1)  “It feels more suited to primary aged children than high school. it felt very childlike” (n=1)  “The Mind your Mate app helping students in well-being of study and life, it's really helpful.” (n=1)  “I thought the information was not useful or applicable enough to help me support friends through their mental health issues. It could have a higher level of information on how to support a friend going through a crisis, and what to say or do to help.” (n=1) | | | | | | |

Open-ended responses from students who did not download the app. At the 6-month survey 18 participants indicated they did not download the app; 33 students did not download the app at the 12-month survey.

| Did you download the Mind your Mate App? No. If no, why not? | T2 | “Not bothered” (n=3)  “I have had no need to download the application.” (n=2)  “Forgot” (n=2)  “Takes up too much storage and data” (n=2)  “We did not have the lesson I believe. Trust me if I had the offer I would've!” (n=2)  “I do not have the time to download it" (n=1)  “Because I didn't feel as though there was anyone I could connect to.” (n=1)  “It seemed like a personal invasion” (n=1) |
| --- | --- | --- |
|  | T3 | “I don't believe it to be necessary for my circumstances." (n=6)  “Couldn’t be bothered” (n=5)  “Forgot” (n=3)  “No storage left on my phone” (n=3)  “I don't believe we were told to as I would've if I was encouraged to.” (n=2)  “because I don't have close enugh relationships with friends to fell comfortable connecting” (n=1)  “No time” (n=1) |
