# Supplementary material for: Evaluating a Peer-Support Mobile App for Mental Health and Substance Use Among Adolescents Over 12 Months During the COVID-19 Pandemic: Randomized Controlled Trial
Source: J Med Internet Res. 2023 Sep 27;25:e45216. doi: 10.2196/45216 (PMC10538359; doi:10.2196/45216)
Supplement: Multimedia Appendix 2 [file jmir_v25i1e45216_app2.pdf]

CONSORT-EHEALTH (V 1.6.1) - Submission/Publication Form

The CONSORT-EHEALTH checklist is intended for authors of randomized trials evaluating web-based and Internet-based applications/interventions, including mobile interventions, electronic games (incl multiplayer games), social media, certain telehealth applications, and other interactive and/or networked electronic applications. Some of the items (e.g. all subitems under item 5 - description of the intervention) may also be applicable for other study designs.

The goal of the CONSORT EHEALTH checklist and guideline is to be
a) a guide for reporting for authors of RCTs,
b) to form a basis for appraisal of an ehealth trial (in terms of validity)

CONSORT-EHEALTH items/subitems are MANDATORY reporting items for studies published in the Journal of Medical Internet Research and other journals / scientific societies endorsing the checklist.

Items numbered 1., 2., 3., 4a., 4b etc are original CONSORT or CONSORT-NPT (non-pharmacologic treatment) items.
Items with Roman numerals (i., ii, iii, iv etc.) are CONSORT-EHEALTH extensions/clarifications.

As the CONSORT-EHEALTH checklist is still considered in a formative stage, we would ask that you also RATE ON A SCALE OF 1-5 how important/useful you feel each item is FOR THE PURPOSE OF THE CHECKLIST and reporting guideline (optional).

Mandatory reporting items are marked with a red *.
In the textboxes, either copy & paste the relevant sections from your manuscript into this form - please include any quotes from your manuscript in QUOTATION MARKS,
or answer directly by providing additional information not in the manuscript, or elaborating on why the item was not relevant for this study.

YOUR ANSWERS WILL BE PUBLISHED AS A SUPPLEMENTARY FILE TO YOUR PUBLICATION IN JMIR AND ARE CONSIDERED PART OF YOUR PUBLICATION (IF ACCEPTED).
Please fill in these questions diligently. Information will not be copyedited, so please use proper spelling and grammar, use correct capitalization, and avoid abbreviations.

DO NOT FORGET TO SAVE AS PDF _AND_ CLICK THE SUBMIT BUTTON SO YOUR ANSWERS ARE IN OUR DATABASE !!!

Citation Suggestion (if you append the pdf as Appendix we suggest to cite this paper in the caption):

Eysenbach G, CONSORT-EHEALTH Group
CONSORT-EHEALTH: Improving and Standardizing Evaluation Reports of Web-based and Mobile Health Interventions
J Med Internet Res 2011;13(4):e126
URL: http://www.jmir.org/2011/4/e126/
doi: 10.2196/jmir.1923
PMID: 22209829

louise.birrell@sydney.edu.au Switch accounts

Not shared

Draft saved

* Indicates required question

Your name *

First Last

Louise Birrell

Primary Affiliation (short), City, Country *

University of Toronto, Toronto, Canada

The Matilda Centre for Research in Mental Hea

Your e-mail address *

abc@gmail.com

louise.birrell@sydney.edu.au

Title of your manuscript *

Provide the (draft) title of your manuscript.

Evaluating a Peer-Support Mobile App for Mental Health and Substance Use Among Adolescents Over 12 Months During the COVID-19 Pandemic: Randomized Controlled Trial

Name of your App/Software/Intervention *

If there is a short and a long/alternate name, write the short name first and add the long name in brackets.

Mind your Mate

Evaluated Version (if any)

e.g. "V1", "Release 2017-03-01", "Version 2.0.27913"

Version 1.0.7

Language(s) *

What language is the intervention/app in? If multiple languages are available, separate by comma (e.g. "English, French")

English

URL of your Intervention Website or App

e.g. a direct link to the mobile app on app in appstore (itunes, Google Play), or URL of the website. If the intervention is a DVD or hardware, you can also link to an Amazon page.

https://www.mindyourmate.org/

URL of an image/screenshot (optional)

Your answer

Accessibility *

Can an enduser access the intervention presently?

- ○ access is free and open
- ○ access only for special usergroups, not open
- ○ access is open to everyone, but requires payment/subscription/in-app purchases
- ○ app/intervention no longer accessible
- ◉ Other: app is free but needs an access code from developers as it is still witl

Primary Medical Indication/Disease/Condition *

e.g. "Stress", "Diabetes", or define the target group in brackets after the condition, e.g. "Autism (Parents of children with)", "Alzheimers (Informal Caregivers of)"

Anxiety, depression, substance misuse

Primary Outcomes measured in trial *

comma-separated list of primary outcomes reported in the trial

Anxiety symptoms, depression symptoms, sub

Secondary/other outcomes

Are there any other outcomes the intervention is expected to affect?

mental health and substance use literacy

Recommended "Dose" *

What do the instructions for users say on how often the app should be used?

- ○ Approximately Daily
- ○ Approximately Weekly
- ○ Approximately Monthly
- ○ Approximately Yearly
- ◉ "as needed"
- ○ Other:

Approx. Percentage of Users (starters) still using the app as recommended after 3 months *

- ○ unknown / not evaluated
- ○ 0-10%
- ○ 11-20%
- ○ 21-30%
- ○ 31-40%
- ○ 41-50%
- ○ 51-60%
- ○ 61-70%
- ○ 71%-80%
- ○ 81-90%
- ○ 91-100%
- ◉ Other: As this was the first trial of the app we did not have a reccomended us

Overall, was the app/intervention effective? *

- ○ yes: all primary outcomes were significantly better in intervention group vs control
- ◉ partly: SOME primary outcomes were significantly better in intervention group vs control
- ○ no statistically significant difference between control and intervention
- ○ potentially harmful: control was significantly better than intervention in one or more outcomes
- ○ inconclusive: more research is needed
- ○ Other:

Article Preparation Status/Stage *

At which stage in your article preparation are you currently (at the time you fill in this form)

- ○ not submitted yet - in early draft status
- ○ not submitted yet - in late draft status, just before submission
- ○ submitted to a journal but not reviewed yet
- ○ submitted to a journal and after receiving initial reviewer comments
- ◉ submitted to a journal and accepted, but not published yet
- ○ published
- ○ Other:

Journal *

If you already know where you will submit this paper (or if it is already submitted), please provide the journal name (if it is not JMIR, provide the journal name under "other")

- ○ not submitted yet / unclear where I will submit this
- ◉ Journal of Medical Internet Research (JMIR)
- ○ JMIR mHealth and UHealth
- ○ JMIR Serious Games
- ○ JMIR Mental Health
- ○ JMIR Public Health
- ○ JMIR Formative Research
- ○ Other JMIR sister journal
- ○ Other:

Is this a full powered effectiveness trial or a pilot/feasibility trial? *

- ◉ Pilot/feasibility
- ○ Fully powered

Manuscript tracking number *

If this is a JMIR submission, please provide the manuscript tracking number under "other" (The ms tracking number can be found in the submission acknowledgement email, or when you login as author in JMIR. If the paper is already published in JMIR, then the ms tracking number is the four-digit number at the end of the DOI, to be found at the bottom of each published article in JMIR)

- ◯ no ms number (yet) / not (yet) submitted to / published in JMIR
- ◉ Other: ms#45216

TITLE AND ABSTRACT

1a) TITLE: Identification as a randomized trial in the title

1a) Does your paper address CONSORT item 1a? *

I.e does the title contain the phrase "Randomized Controlled Trial"? (if not, explain the reason under "other")

- ◉ yes
- ◯ Other:

1a-i) Identify the mode of delivery in the title

Identify the mode of delivery. Preferably use “web-based” and/or “mobile” and/or “electronic game” in the title. Avoid ambiguous terms like “online”, “virtual”, “interactive”. Use “Internet-based” only if Intervention includes non-web-based Internet components (e.g. email), use “computer-based” or “electronic” only if offline products are used. Use “virtual” only in the context of “virtual reality” (3-D worlds). Use “online” only in the context of “online support groups”. Complement or substitute product names with broader terms for the class of products (such as “mobile” or “smart phone” instead of “iphone”), especially if the application runs on different platforms.

	1	2	3	4	5	
subitem not at all important	○	○	○	○	○	essential

Does your paper address subitem 1a-i? *

Copy and paste relevant sections from manuscript title (include quotes in quotation marks "like this" to indicate direct quotes from your manuscript), or elaborate on this item by providing additional information not in the ms, or briefly explain why the item is not applicable/relevant for your study

"Evaluating a Peer-Support Mobile App for Mental Health and Substance Use Among Adolescents During the COVID-19 Pandemic: 12-Month Outcomes of a Randomized Controlled Trial"

1a-ii) Non-web-based components or important co-interventions in title

Mention non-web-based components or important co-interventions in title, if any (e.g., “with telephone support”).

	1	2	3	4	5	
subitem not at all important	○	○	○	○	○	essential

Does your paper address subitem 1a-ii?

Copy and paste relevant sections from manuscript title (include quotes in quotation marks "like this" to indicate direct quotes from your manuscript), or elaborate on this item by providing additional information not in the ms, or briefly explain why the item is not applicable/relevant for your study

I could not fit this additional information within the title

1a-iii) Primary condition or target group in the title

Mention primary condition or target group in the title, if any (e.g., "for children with Type I Diabetes") Example: A Web-based and Mobile Intervention with Telephone Support for Children with Type I Diabetes: Randomized Controlled Trial

	1	2	3	4	5	
subitem not at all important	○	○	○	○	○	essential

Does your paper address subitem 1a-iii? *

Copy and paste relevant sections from manuscript title (include quotes in quotation marks "like this" to indicate direct quotes from your manuscript), or elaborate on this item by providing additional information not in the ms, or briefly explain why the item is not applicable/relevant for your study

"Evaluating a peer-support mobile app for mental health and substance use among adolescents during the COVID-19 pandemic: 12-month RCT outcomes"

1b) ABSTRACT: Structured summary of trial design, methods, results, and conclusions

NPT extension: Description of experimental treatment, comparator, care providers, centers, and blinding status.

1b-i) Key features/functionalities/components of the intervention and comparator in the METHODS section of the ABSTRACT

Mention key features/functionalities/components of the intervention and comparator in the abstract. If possible, also mention theories and principles used for designing the site. Keep in mind the needs of systematic reviewers and indexers by including important synonyms. (Note: Only report in the abstract what the main paper is reporting. If this information is missing from the main body of text, consider adding it)

	1	2	3	4	5	
subitem not at all important	○	○	○	○	○	essential

Does your paper address subitem 1b-i? *

Copy and paste relevant sections from the manuscript abstract (include quotes in quotation marks "like this" to indicate direct quotes from your manuscript), or elaborate on this item by providing additional information not in the ms, or briefly explain why the item is not applicable/relevant for your study

"To evaluate the efficacy of the Mind your Mate program, a digital peer support program, in improving mental health symptoms, reducing the uptake of substance use, and increasing help-seeking. The Mind your Mate program consists of an online 40-minute classroom lesson and a companion smartphone mobile application (app). The active control group received school-based health education as usual."

1b-ii) Level of human involvement in the METHODS section of the ABSTRACT

Clarify the level of human involvement in the abstract, e.g., use phrases like “fully automated” vs. “therapist/nurse/care provider/physician-assisted” (mention number and expertise of providers involved, if any). (Note: Only report in the abstract what the main paper is reporting. If this information is missing from the main body of text, consider adding it)

	1	2	3	4	5	
subitem not at all important	○	●	○	○	○	essential

Clear selection

Does your paper address subitem 1b-ii?

Copy and paste relevant sections from the manuscript abstract (include quotes in quotation marks "like this" to indicate direct quotes from your manuscript), or elaborate on this item by providing additional information not in the ms, or briefly explain why the item is not applicable/relevant for your study

"The Mind your Mate program consists of an online 40-minute classroom lesson and a companion smartphone mobile application (app). The active control group received school-based health education as usual."

1b-iii) Open vs. closed, web-based (self-assessment) vs. face-to-face assessments in the METHODS section of the ABSTRACT

Mention how participants were recruited (online vs. offline), e.g., from an open access website or from a clinic or a closed online user group (closed usergroup trial), and clarify if this was a purely web-based trial, or there were face-to-face components (as part of the intervention or for assessment). Clearly say if outcomes were self-assessed through questionnaires (as common in web-based trials). Note: In traditional offline trials, an open trial (open-label trial) is a type of clinical trial in which both the researchers and participants know which treatment is being administered. To avoid confusion, use “blinded” or “unblinded” to indicated the level of blinding instead of “open”, as “open” in web-based trials usually refers to “open access” (i.e. participants can self-enrol). (Note: Only report in the abstract what the main paper is reporting. If this information is missing from the main body of text, consider adding it)

	1	2	3	4	5	
subitem not at all important	○	○	○	○	○	essential

Does your paper address subitem 1b-iii?

Copy and paste relevant sections from the manuscript abstract (include quotes in quotation marks "like this" to indicate direct quotes from your manuscript), or elaborate on this item by providing additional information not in the ms, or briefly explain why the item is not applicable/relevant for your study

"A cluster randomized controlled trial (RCT) was conducted with 12 secondary schools and 166 students (mean age=15.3 years, SD=0.41, 46% female, 80% born in Australia)"

1b-iv) RESULTS section in abstract must contain use data

Report number of participants enrolled/assessed in each group, the use/uptake of the intervention (e.g., attrition/adherence metrics, use over time, number of logins etc.), in addition to primary/secondary outcomes. (Note: Only report in the abstract what the main paper is reporting. If this information is missing from the main body of text, consider adding it)

	1	2	3	4	5	
subitem not at all important	○	○	○	○	○	essential

Does your paper address subitem 1b-iv?

Copy and paste relevant sections from the manuscript abstract (include quotes in quotation marks "like this" to indicate direct quotes from your manuscript), or elaborate on this item by providing additional information not in the ms, or briefly explain why the item is not applicable/relevant for your study

"Students who received the Mind your Mate program had reduced growth in depressive symptoms over a 12-month period, compared to controls (b=-1.86, 95% CI=3.73 – 0.02)"

1b-v) CONCLUSIONS/DISCUSSION in abstract for negative trials

Conclusions/Discussions in abstract for negative trials: Discuss the primary outcome - if the trial is negative (primary outcome not changed), and the intervention was not used, discuss whether negative results are attributable to lack of uptake and discuss reasons. (Note: Only report in the abstract what the main paper is reporting. If this information is missing from the main body of text, consider adding it)

	1	2	3	4	5	
subitem not at all important	○	◉	○	○	○	essential

Clear selection

Does your paper address subitem 1b-v?

Copy and paste relevant sections from the manuscript abstract (include quotes in quotation marks "like this" to indicate direct quotes from your manuscript), or elaborate on this item by providing additional information not in the ms, or briefly explain why the item is not applicable/relevant for your study

"A novel digital health intervention, the Mind your Mate program, reduced depression symptoms in adolescents over 12-months compared to a control condition."

INTRODUCTION

2a) In INTRODUCTION: Scientific background and explanation of rationale

2a-i) Problem and the type of system/solution

Describe the problem and the type of system/solution that is object of the study: intended as stand-alone intervention vs. incorporated in broader health care program? Intended for a particular patient population? Goals of the intervention, e.g., being more cost-effective to other interventions, replace or complement other solutions? (Note: Details about the intervention are provided in “Methods” under 5)

	1	2	3	4	5	
subitem not at all important	○	○	○	○	○	essential

Does your paper address subitem 2a-i? *

Copy and paste relevant sections from the manuscript (include quotes in quotation marks "like this" to indicate direct quotes from your manuscript), or elaborate on this item by providing additional information not in the ms, or briefly explain why the item is not applicable/relevant for your study

"While mental health apps and online programs have proliferated, there is a lack of evidence-based interventions aiming to upskill adolescents around supporting peers in relation to mental health and/or substance use."

"To evaluate the efficacy of the Mind your Mate program through a cluster randomized controlled trial (RCT) conducted in partnership with Australian secondary schools. We hypothesized that the Mind your Mate intervention would be more effective than active control in;
i) delaying the uptake of alcohol and other drugs
ii) reducing anxiety and depression symptoms
iii) increasing knowledge about mental health, alcohol, and other drugs, and
iv) increasing help-seeking behaviour."

2a-ii) Scientific background, rationale: What is known about the (type of) system

Scientific background, rationale: What is known about the (type of) system that is the object of the study (be sure to discuss the use of similar systems for other conditions/diagnoses, if appropiate), motivation for the study, i.e. what are the reasons for and what is the context for this specific study, from which stakeholder viewpoint is the study performed, potential impact of findings [2]. Briefly justify the choice of the comparator.

	1	2	3	4	5	
subitem not at all important	○	○	○	○	○	essential

Does your paper address subitem 2a-ii? *

Copy and paste relevant sections from the manuscript (include quotes in quotation marks "like this" to indicate direct quotes from your manuscript), or elaborate on this item by providing additional information not in the ms, or briefly explain why the item is not applicable/relevant for your study

Yes - this is addressed in the introduction paragraphs. "Youth mental health has been identified as a global priority. It is estimated the global prevalence of mental disorders among adolescents and children is almost 14% [1] with mental disorders being the largest contributors to global burden of disease in terms of years lived with disability [2]. They result in significant impact on functioning, schooling, health and wellbeing with huge economic costs [3]. Concerningly, common disorders such as depression and anxiety, are on the rise among recent cohorts of young people [4], with the COVID-19 pandemic worsening the impact [5]. Many young people also continue to use and misuse alcohol and other drugs [6], with mental disorders a substantive risk factor for the development of problematic substance use [7].

Mental and substance use disorders typically first onset between adolescence and early adulthood, yet most young people will not seek professional help [8]. Rather, adolescents commonly turn to their friends when experiencing mental health problems [9] and more generally, when facing a significant issue in their lives [10]. In a large survey of over 6,500 young people, friends were the most commonly cited source of help for young people generally, and for young people experiencing high levels of psychological distress [11]. Interventions promoting social support and connection have been identified as an important target for mental health prevention [12]. Yet it is not clear that young people are adequately equipped to provide evidence-based and effective social support to their peers in relation to mental and substance use disorders. A systematic review of existing school-based peer interventions showed that despite the widespread use of peer interventions, there is a lack of evidence demonstrating mental health improvements [13]. There is a clear need to explore novel prevention initiatives providing evidence-based tools young people can access to support peers. Such programs must be easily accessible, engaging and meet young people where they are.

To be engaging and effective, interventions should be delivered in modes relevant to adolescents, taking into account the unequivocal access to digital technologies recent cohorts of adolescents have. In Western countries such as the US, UK and Australia 90-95% of teenagers own a mobile phone, nearly all of which being internet connected smartphones [14-16]. One key opportunity for the prevention of mental disorders is harnessing mobile and online technology to deliver easily accessible prevention that can be widely disseminated at relatively low cost, in a format that is acceptable and engaging to adolescents. The COVID-19 pandemic initiated a paradigm shift accelerating the uptake of digital technologies to deliver scalable, accessible and affordable mental health care [17]. While mental health apps and online programs have proliferated, there is a lack of evidence-based interventions aiming to upskill adolescents around supporting peers in relation to mental health and/or substance use.

The Mind your Mate program, a classroom lesson and mobile application (app), aims to upskill and empower adolescents to better support their peers. Adolescents complete one online lesson in class that introduces the concept of supporting peers, and mental health and substance use literacy. Students are prompted to download and register on the Mind your Mate smartphone app at the end of the lesson. The app is a self-guided program that (builds upon the concepts introduced in the classroom lesson) by provides normative information about mental health and substance use, skill building modules for peer-support [illegible]

(including active listening and checking in), self-care modules as well as referral and support options to encourage further help-seeking. Full details about the app development process and app features/functionality have been previously published [18, 19]. The next step is to evaluate the effectiveness of the program in improving youth mental health."

"Active Control Group

Schools allocated to the control condition were asked to implement their usual health and physical education classes for the 12-month trial period. The year 9-10 Health and Physical Education curriculum mandates that wellbeing and alcohol and other drug use content be taught. As such, all control schools delivered curriculum-based health education during the trial ranging from 1 to 15 mental health lessons and 2 to 21 lessons about alcohol and other drugs. Control schools were offered access to the intervention at the completion of the trial."

2b) In INTRODUCTION: Specific objectives or hypotheses

Does your paper address CONSORT subitem 2b? *

Copy and paste relevant sections from the manuscript (include quotes in quotation marks "like this" to indicate direct quotes from your manuscript), or elaborate on this item by providing additional information not in the ms, or briefly explain why the item is not applicable/relevant for your study

"Aims
To evaluate the efficacy of the Mind your Mate program through a cluster randomized controlled trial (RCT) conducted in partnership with Australian secondary schools. We hypothesized that the Mind your Mate intervention would be more effective than active control in;
i) delaying the uptake of alcohol and other drugs
ii) reducing anxiety and depression symptoms
iii) increasing knowledge about mental health, alcohol, and other drugs, and
iv) increasing help-seeking behaviour."

METHODS

3a) Description of trial design (such as parallel, factorial) including allocation ratio

Does your paper address CONSORT subitem 3a? *

Copy and paste relevant sections from the manuscript (include quotes in quotation marks "like this" to indicate direct quotes from your manuscript), or elaborate on this item by providing additional information not in the ms, or briefly explain why the item is not applicable/relevant for your study

"A cluster RCT was conducted in 12 independent and public secondary schools in the Greater Sydney region of New South Wales (NSW). Schools were randomly allocated to one of two parallel arms: the Mind your Mate condition or the active control condition (health education as usual). Cluster randomisation was used to avoid contamination of the control condition by the intervention through student communication. All year 9 students (approximately 14 years old) at participating schools were invited to participate."

3b) Important changes to methods after trial commencement (such as eligibility criteria), with reasons

Does your paper address CONSORT subitem 3b? *

Copy and paste relevant sections from the manuscript (include quotes in quotation marks "like this" to indicate direct quotes from your manuscript), or elaborate on this item by providing additional information not in the ms, or briefly explain why the item is not applicable/relevant for your study

N/A no significant changes were made after trial commencement.

3b-i) Bug fixes, Downtimes, Content Changes

Bug fixes, Downtimes, Content Changes: ehealth systems are often dynamic systems. A description of changes to methods therefore also includes important changes made on the intervention or comparator during the trial (e.g., major bug fixes or changes in the functionality or content) (5-iii) and other “unexpected events” that may have influenced study design such as staff changes, system failures/downtimes, etc. [2].

	1	2	3	4	5	
subitem not at all important	○	○	○	○	○	essential

Does your paper address subitem 3b-i?

Copy and paste relevant sections from the manuscript (include quotes in quotation marks "like this" to indicate direct quotes from your manuscript), or elaborate on this item by providing additional information not in the ms, or briefly explain why the item is not applicable/relevant for your study

No as we did not make changes to the app during the trial.

4a) Eligibility criteria for participants

Does your paper address CONSORT subitem 4a? *

Copy and paste relevant sections from the manuscript (include quotes in quotation marks "like this" to indicate direct quotes from your manuscript), or elaborate on this item by providing additional information not in the ms, or briefly explain why the item is not applicable/relevant for your study

"Students were eligible to participate in the trial if they received parental consent, provided active consent owned10-4 a smartphone."

4a-i) Computer / Internet literacy

Computer / Internet literacy is often an implicit “de facto” eligibility criterion - this should be explicitly clarified.

	1	2	3	4	5	
subitem not at all important	○	○	○	○	○	essential

Does your paper address subitem 4a-i?

Copy and paste relevant sections from the manuscript (include quotes in quotation marks "like this" to indicate direct quotes from your manuscript), or elaborate on this item by providing additional information not in the ms, or briefly explain why the item is not applicable/relevant for your study

"Students were eligible to participate in the trial if they received parental consent, provided active consent owned a smartphone."

4a-ii) Open vs. closed, web-based vs. face-to-face assessments:

Open vs. closed, web-based vs. face-to-face assessments: Mention how participants were recruited (online vs. offline), e.g., from an open access website or from a clinic, and clarify if this was a purely web-based trial, or there were face-to-face components (as part of the intervention or for assessment), i.e., to what degree got the study team to know the participant. In online-only trials, clarify if participants were quasi-anonymous and whether having multiple identities was possible or whether technical or logistical measures (e.g., cookies, email confirmation, phone calls) were used to detect/prevent these.

	1	2	3	4	5	
subitem not at all important	○	○	○	○	○	essential

Does your paper address subitem 4a-ii? *

Copy and paste relevant sections from the manuscript (include quotes in quotation marks "like this" to indicate direct quotes from your manuscript), or elaborate on this item by providing additional information not in the ms, or briefly explain why the item is not applicable/relevant for your study

"Recruitment took place between June 2020 to March 2021. A random selection of 126 independent and public schools located within the Greater Sydney Area of NSW were contacted using publicly available details. The study was also advertised through the researchers' networks.

School principals and relevant school staff members (e.g. Head of Wellbeing) were initially approached using an invitation email outlining the aims of the study and inviting their school to take part. The research team followed up with staff via phone calls and emails. Principals who agreed for their school to take part were asked to identify an appropriate staff member (e.g. head of student wellbeing or health and physical education coordinator), who became the point of contact for study coordination."

"Baseline data collection was conducted between November 2020 and May 2021, with students completing follow-up surveys at 6- and 12-months post baseline (final follow-up data collected by May 2022). All students (intervention and control conditions) who gave consent and had parental consent were asked to complete the web-based self-report surveys in a supervised classroom setting. Students were assigned a unique participant identifier generated in REDCap (Research Electronic Data Capture; Vanderbilt University), a secure web-based data collection system. This identifier was used to email students personalized survey links. The unique code linked student data over time while maintaining confidentiality. Absent students were contacted by the research team (using details provided upon registration) and asked to complete the survey at home in their own time. Students who had left participating schools were contacted using secondary email addresses provided at baseline. Students were reimbursed for their participation with entry into a draw for 1 of 5 AU$50 (US $34) Prezzee gift vouchers at each survey occasion."

4a-iii) Information giving during recruitment

Information given during recruitment. Specify how participants were briefed for recruitment and in the informed consent procedures (e.g., publish the informed consent documentation as appendix, see also item X26), as this information may have an effect on user self-selection, user expectation and may also bias results.

	1	2	3	4	5	
subitem not at all important	○	○	○	○	○	essential

Does your paper address subitem 4a-iii?

Copy and paste relevant sections from the manuscript (include quotes in quotation marks "like this" to indicate direct quotes from your manuscript), or elaborate on this item by providing additional information not in the ms, or briefly explain why the item is not applicable/relevant for your study

"Recruitment took place between June 2020 to March 2021. A random selection of 126 independent and public schools located within the Greater Sydney Area of NSW were contacted using publicly available details. The study was also advertised through the researchers' networks.

School principals and relevant school staff members (e.g. Head of Wellbeing) were initially approached using an invitation email outlining the aims of the study and inviting their school to take part. The research team followed up with staff via phone calls and emails. Principals who agreed for their school to take part were asked to identify an appropriate staff member (e.g. head of student wellbeing or health and physical education coordinator), who became the point of contact for study coordination.

Opt-in parental and guardian information and consent forms were sent electronically or via hardcopy to all parents or guardians of the selected year group. Students were eligible to participate in the trial if they received parental consent, provided active consent owned10-4 a smartphone. "

4b) Settings and locations where the data were collected

Does your paper address CONSORT subitem 4b? *

Copy and paste relevant sections from the manuscript (include quotes in quotation marks "like this" to indicate direct quotes from your manuscript), or elaborate on this item by providing additional information not in the ms, or briefly explain why the item is not applicable/relevant for your study

"All students (intervention and control conditions) who gave consent and had parental consent were asked to complete the web-based self-report surveys in a supervised classroom setting."

4b-i) Report if outcomes were (self-)assessed through online questionnaires

Clearly report if outcomes were (self-)assessed through online questionnaires (as common in web-based trials) or otherwise.

	1	2	3	4	5	
subitem not at all important	○	○	○	○	○	essential

Does your paper address subitem 4b-i? *

Copy and paste relevant sections from the manuscript (include quotes in quotation marks "like this" to indicate direct quotes from your manuscript), or elaborate on this item by providing additional information not in the ms, or briefly explain why the item is not applicable/relevant for your study

"All students (intervention and control conditions) who gave consent and had parental consent were asked to complete the web-based self-report surveys in a supervised classroom setting."

4b-ii) Report how institutional affiliations are displayed

Report how institutional affiliations are displayed to potential participants [on ehealth media], as affiliations with prestigious hospitals or universities may affect volunteer rates, use, and reactions with regards to an intervention.(Not a required item – describe only if this may bias results)

	1	2	3	4	5	
subitem not at all important	○	○	○	○	○	essential

Does your paper address subitem 4b-ii?

Copy and paste relevant sections from the manuscript (include quotes in quotation marks "like this" to indicate direct quotes from your manuscript), or elaborate on this item by providing additional information not in the ms, or briefly explain why the item is not applicable/relevant for your study

This is not applicable, as participants were recruited at the school level and took part in the trial if their school agreed and they then provided individual and parental consent.

5) The interventions for each group with sufficient details to allow replication, including how and when they were actually administered

5-i) Mention names, credential, affiliations of the developers, sponsors, and owners

Mention names, credential, affiliations of the developers, sponsors, and owners [6] (if authors/evaluators are owners or developer of the software, this needs to be declared in a “Conflict of interest” section or mentioned elsewhere in the manuscript).

	1	2	3	4	5	
subitem not at all important	○	○	○	○	○	essential

Does your paper address subitem 5-i?

Copy and paste relevant sections from the manuscript (include quotes in quotation marks "like this" to indicate direct quotes from your manuscript), or elaborate on this item by providing additional information not in the ms, or briefly explain why the item is not applicable/relevant for your study

"Acknowledgements
The authors would like to acknowledge Netfront Pty Ltd for their IT support on and app development work. We would also like to acknowledge input from the Youth Advisory Board members of the NHMRC Centre of Research Excellence in the Prevention and Early Intervention in Mental and Substance Use Disorders (PREMISE).

Conflicts of Interest
Dr Birrell the developed of the Mind your Mate program."

5-ii) Describe the history/development process

Describe the history/development process of the application and previous formative evaluations (e.g., focus groups, usability testing), as these will have an impact on adoption/use rates and help with interpreting results.

	1	2	3	4	5	
subitem not at all important	○	○	○	○	○	essential

Does your paper address subitem 5-ii?

Copy and paste relevant sections from the manuscript (include quotes in quotation marks "like this" to indicate direct quotes from your manuscript), or elaborate on this item by providing additional information not in the ms, or briefly explain why the item is not applicable/relevant for your study

The development process has been detailed in a separate publication, which is referenced in the current manuscript. "Full details about the app development process and app features/functionality have been previously published [18, 19]. "

5-iii) Revisions and updating

Revisions and updating. Clearly mention the date and/or version number of the application/intervention (and comparator, if applicable) evaluated, or describe whether the intervention underwent major changes during the evaluation process, or whether the development and/or content was “frozen” during the trial. Describe dynamic components such as news feeds or changing content which may have an impact on the replicability of the intervention (for unexpected events see item 3b).

	1	2	3	4	5	
subitem not at all important	○	○	○	○	○	essential

Does your paper address subitem 5-iii?

Copy and paste relevant sections from the manuscript (include quotes in quotation marks "like this" to indicate direct quotes from your manuscript), or elaborate on this item by providing additional information not in the ms, or briefly explain why the item is not applicable/relevant for your study

NA. We did not have any dynamic elements and did not update the app during the trial.

5-iv) Quality assurance methods

Provide information on quality assurance methods to ensure accuracy and quality of information provided [1], if applicable.

	1	2	3	4	5	
subitem not at all important	○	○	○	○	○	essential

Does your paper address subitem 5-iv?

Copy and paste relevant sections from the manuscript (include quotes in quotation marks "like this" to indicate direct quotes from your manuscript), or elaborate on this item by providing additional information not in the ms, or briefly explain why the item is not applicable/relevant for your study

This information is detailed in the development paper, which is referenced in the current manuscript: "Full details about the app development process and app features/functionality have been previously published [18, 19]. "

5-v) Ensure replicability by publishing the source code, and/or providing screenshots/screen-capture video, and/or providing flowcharts of the algorithms used

Ensure replicability by publishing the source code, and/or providing screenshots/screen-capture video, and/or providing flowcharts of the algorithms used. Replicability (i.e., other researchers should in principle be able to replicate the study) is a hallmark of scientific reporting.

	1	2	3	4	5	
subitem not at all important	○	○	○	○	○	essential

Does your paper address subitem 5-v?

Copy and paste relevant sections from the manuscript (include quotes in quotation marks "like this" to indicate direct quotes from your manuscript), or elaborate on this item by providing additional information not in the ms, or briefly explain why the item is not applicable/relevant for your study

Screenshots of the intervention as included in the manuscript on page 8.

5-vi) Digital preservation

Digital preservation: Provide the URL of the application, but as the intervention is likely to change or disappear over the course of the years; also make sure the intervention is archived (Internet Archive, webcitation.org, and/or publishing the source code or screenshots/videos alongside the article). As pages behind login screens cannot be archived, consider creating demo pages which are accessible without login.

	1	2	3	4	5	
subitem not at all important	○	○	○	○	○	essential

Does your paper address subitem 5-vi?

Copy and paste relevant sections from the manuscript (include quotes in quotation marks "like this" to indicate direct quotes from your manuscript), or elaborate on this item by providing additional information not in the ms, or briefly explain why the item is not applicable/relevant for your study

Screenshots of the intervention as included in the manuscript on page 8.

5-vii) Access

Access: Describe how participants accessed the application, in what setting/context, if they had to pay (or were paid) or not, whether they had to be a member of specific group. If known, describe how participants obtained “access to the platform and Internet” [1]. To ensure access for editors/reviewers/readers, consider to provide a “backdoor” login account or demo mode for reviewers/readers to explore the application (also important for archiving purposes, see vi).

	1	2	3	4	5	
subitem not at all important	○	○	○	○	○	essential

Does your paper address subitem 5-vii? *

Copy and paste relevant sections from the manuscript (include quotes in quotation marks "like this" to indicate direct quotes from your manuscript), or elaborate on this item by providing additional information not in the ms, or briefly explain why the item is not applicable/relevant for your study

"The Mind your Mate classroom lesson is a web-based educational resource facilitated by teachers at the participating schools, delivered through the study website. Teachers were given an implementation guide with instructions on how students could access the classroom lesson, and an outline of the links to the NSW Personal Development, Health, and Physical Education Syllabus. The lesson ran for approximately 40 minutes, and contained animated examples, quizzes, and interactive activities introducing the concept of supporting peers, mental health and substance use literacy, help-seeking and harm-minimisation information in relation to substance use. At the completion of the lesson, a video introduced the Mind your Mate app, demonstrated its features and directing students to download the app onto their mobile phone. Students were asked to use the mobile app as needed in a self-directed way over the following 12 months."

"The app was designed to be self-guided and was only accessible to students attending schools allocated to the intervention group (i.e., a school-specific code was required to access the app). "

5-viii) Mode of delivery, features/functionalities/components of the intervention and comparator, and the theoretical framework

Describe mode of delivery, features/functionalities/components of the intervention and comparator, and the theoretical framework [6] used to design them (instructional strategy [1], behaviour change techniques, persuasive features, etc., see e.g., [7, 8] for terminology). This includes an in-depth description of the content (including where it is coming from and who developed it) [1],” whether [and how] it is tailored to individual circumstances and allows users to track their progress and receive feedback” [6]. This also includes a description of communication delivery channels and – if computer-mediated communication is a component – whether communication was synchronous or asynchronous [6]. It also includes information on presentation strategies [1], including page design principles, average amount of text on pages, presence of hyperlinks to other resources, etc. [1].

	1	2	3	4	5	
subitem not at all important	○	○	○	○	○	essential

Does your paper address subitem 5-viii? *

Copy and paste relevant sections from the manuscript (include quotes in quotation marks "like this" to indicate direct quotes from your manuscript), or elaborate on this item by providing additional information not in the ms, or briefly explain why the item is not applicable/relevant for your study

This information is detailed in the development paper, which is referenced in the current manuscript: "Full details about the app development process and app features/functionality have been previously published [18, 19]. "

5-ix) Describe use parameters

Describe use parameters (e.g., intended “doses” and optimal timing for use). Clarify what instructions or recommendations were given to the user, e.g., regarding timing, frequency, heaviness of use, if any, or was the intervention used ad libitum.

	1	2	3	4	5	
subitem not at all important	○	○	○	○	○	essential

Does your paper address subitem 5-ix?

Copy and paste relevant sections from the manuscript (include quotes in quotation marks "like this" to indicate direct quotes from your manuscript), or elaborate on this item by providing additional information not in the ms, or briefly explain why the item is not applicable/relevant for your study

"At the completion of the lesson, a video introduced the Mind your Mate app, demonstrated its features and directing students to download the app onto their mobile phone. Students were asked to use the mobile app as needed in a self-directed way over the following 12 months."

5-x) Clarify the level of human involvement

Clarify the level of human involvement (care providers or health professionals, also technical assistance) in the e-intervention or as co-intervention (detail number and expertise of professionals involved, if any, as well as “type of assistance offered, the timing and frequency of the support, how it is initiated, and the medium by which the assistance is delivered”. It may be necessary to distinguish between the level of human involvement required for the trial, and the level of human involvement required for a routine application outside of a RCT setting (discuss under item 21 – generalizability).

	1	2	3	4	5	
subitem not at all important	○	○	○	○	○	essential

Does your paper address subitem 5-x?

Copy and paste relevant sections from the manuscript (include quotes in quotation marks "like this" to indicate direct quotes from your manuscript), or elaborate on this item by providing additional information not in the ms, or briefly explain why the item is not applicable/relevant for your study

"The Mind your Mate classroom lesson is a web-based educational resource facilitated by teachers at the participating schools, delivered through the study website. Teachers were given an implementation guide with instructions on how students could access the classroom lesson, and an outline of the links to the NSW Personal Development, Health, and Physical Education Syllabus. The lesson ran for approximately 40 minutes, and contained animated examples, quizzes, and interactive activities introducing the concept of supporting peers, mental health and substance use literacy, help-seeking and harm-minimisation information in relation to substance use. At the completion of the lesson, a video introduced the Mind your Mate app, demonstrated its features and directing students to download the app onto their mobile phone. Students were asked to use the mobile app as needed in a self-directed way over the following 12 months."

5-xi) Report any prompts/reminders used

Report any prompts/reminders used: Clarify if there were prompts (letters, emails, phone calls, SMS) to use the application, what triggered them, frequency etc. It may be necessary to distinguish between the level of prompts/reminders required for the trial, and the level of prompts/reminders for a routine application outside of a RCT setting (discuss under item 21 – generalizability).

	1	2	3	4	5	
subitem not at all important	○	○	○	○	○	essential

Does your paper address subitem 5-xi? *

Copy and paste relevant sections from the manuscript (include quotes in quotation marks "like this" to indicate direct quotes from your manuscript), or elaborate on this item by providing additional information not in the ms, or briefly explain why the item is not applicable/relevant for your study

"Two pop-up notification reminders were also sent to those who downloaded the app in a 2.5-week period around the 6-month follow-up"

5-xii) Describe any co-interventions (incl. training/support)

Describe any co-interventions (incl. training/support): Clearly state any interventions that are provided in addition to the targeted eHealth intervention, as ehealth intervention may not be designed as stand-alone intervention. This includes training sessions and support [1]. It may be necessary to distinguish between the level of training required for the trial, and the level of training for a routine application outside of a RCT setting (discuss under item 21 – generalizability.

	1	2	3	4	5	
subitem not at all important	○	○	○	○	○	essential

Does your paper address subitem 5-xii? *

Copy and paste relevant sections from the manuscript (include quotes in quotation marks "like this" to indicate direct quotes from your manuscript), or elaborate on this item by providing additional information not in the ms, or briefly explain why the item is not applicable/relevant for your study

"The Mind your Mate classroom lesson is a web-based educational resource facilitated by teachers at the participating schools, delivered through the study website. Teachers were given an implementation guide with instructions on how students could access the classroom lesson, and an outline of the links to the NSW Personal Development, Health, and Physical Education Syllabus. The lesson ran for approximately 40 minutes, and contained animated examples, quizzes, and interactive activities introducing the concept of supporting peers, mental health and substance use literacy, help-seeking and harm-minimisation information in relation to substance use. At the completion of the lesson, a video introduced the Mind your Mate app, demonstrated its features and directing students to download the app onto their mobile phone. Students were asked to use the mobile app as needed in a self-directed way over the following 12 months."

6a) Completely defined pre-specified primary and secondary outcome measures, including how and when they were assessed

Does your paper address CONSORT subitem 6a? *

Copy and paste relevant sections from the manuscript (include quotes in quotation marks "like this" to indicate direct quotes from your manuscript), or elaborate on this item by providing additional information not in the ms, or briefly explain why the item is not applicable/relevant for your study

All outcomes were prespecified in the RCT protocol paper.

"The full study protocol was prospectively published [18]."

"Measures

Mental health and substance use literacy

Mental health and substance use literacy was measured by summing responses from a 12-item knowledge scale developed to measure topics covered by the program. Participants responded true/false to items such as ‘the Australian guidelines say that for people under the age of 18, the safest option is to not drink alcohol at all’ and ‘a pounding heart, feeling sick and sweating can all be symptoms of anxiety’. Higher scores reflected greater mental health and substance use literacy.

Alcohol and Other Substance Use

Frequency of alcohol use was assessed using an adapted version of the Patterns of Alcohol Index [20]. Participants were asked “how often did you have a standard alcoholic drink of any kind in the past 6 months?” (Full drink). To aid accurate reporting, a standard drinks chart was presented with these items.

Other drug use was assessed via items from the Australian Institute of Health and Welfare: National Drug Strategy Household Survey [21]. Students were asked to report ‘yes I have tried’ or ‘no I have never tried’ in relation to the following drugs: tobacco, vaping, cannabis, ecstasy, cocaine, methamphetamines, nonmedical use of prescription drugs, or a combination of drugs at the same time. A composite total score was calculated across all drug types with 1= have tried at least one drug and 0=have never tried drugs.

Mental Health Measures

Anxiety symptoms in the past two weeks were assessed using the Generalized Anxiety Disorder 7-item scale [22], while depression symptoms over the past two weeks were assessed using the Patient Health Questionnaire Adolescent version [23]. General psychological distress in the past month was measured using the Kessler 6 scale [24]. Higher scores reflect higher levels of psychological symptoms.

Help-Seeking Measures

An item from the General Help-Seeking Questionnaire assessed future help-seeking intentions from a friend [25]. Using a 7-point scale, students rated the likelihood of seeking support from a friend for an emotional problem. To aid interpretability this item was dichotomised yes/no, with response options ranging from ‘extremely unlikely’ to ‘unlikely’ coded no, while options from ‘likely’ to ‘extremely’ likely were coded yes. Participants were also asked to indicate whether they sought help from a friend (not related to you) in the past 2 weeks. This item is from the Actual Help-Seeking Questionnaire [26]. "

6a-i) Online questionnaires: describe if they were validated for online use and apply CHERRIES items to describe how the questionnaires were designed/deployed

If outcomes were obtained through online questionnaires, describe if they were validated for online use and apply CHERRIES items to describe how the questionnaires were designed/deployed [9].

	1	2	3	4	5	
subitem not at all important	○	○	○	○	○	essential

Does your paper address subitem 6a-i?

Copy and paste relevant sections from manuscript text

All measures are validated scales and have been previously used in online surveys.

6a-ii) Describe whether and how "use" (including intensity of use/dosage) was defined/measured/monitored

Describe whether and how "use" (including intensity of use/dosage) was defined/measured/monitored (logins, logfile analysis, etc.). Use/adoption metrics are important process outcomes that should be reported in any ehealth trial.

	1	2	3	4	5	
subitem not at all important	○	○	○	○	○	essential

Does your paper address subitem 6a-ii?

Copy and paste relevant sections from manuscript text

"Uptake and adherence

Of the 88 students provided access to the Mind your Mate app, 43 downloaded and registered an account on the mobile app (48.9%). These students accessed the app between 1 and 7 times, opening it an average of 1.7 times (SD=1.6). A mean of 5.2 days (SD=12.6) passed between the first- and last-time students opened the app. The maximum number of days between the first and last time a student used the app was 48 days.

Data was collected on the use of key functions in the app, with the educational modules being the most popular function accessed. Of the registered users, 32.5% students opened at least one and up to 4 different educational modules (n=14/43). The mean number of modules opened by a student was 1.57 modules (SD=0.9), with 5 students opening more than one module. The mood tracker function was used by 23.3% of registered users (n=10/43). These students logged at least one mood entry, with an average of 1.3 mood entries (SD=0.5). In total, 12 total entries were made in the mood tracker. The friend function was used by 8 students to create 9 friend profiles. 18.6% of registered users (n=8/43) created a profile about a friend, with one student making two friend profiles. The conversation planning function and the self-care activity function were not used by any students in the trial."

6a-iii) Describe whether, how, and when qualitative feedback from participants was obtained

Describe whether, how, and when qualitative feedback from participants was obtained (e.g., through emails, feedback forms, interviews, focus groups).

	1	2	3	4	5	
subitem not at all important	○	○	○	○	○	essential

Does your paper address subitem 6a-iii?

Copy and paste relevant sections from manuscript text

"Acceptability
Acceptability analysis measured a subset of intervention participants who met the following criteria: (1) completed the app evaluation section of the surveys and (2) indicated that they downloaded the app. Twenty-five participants at the 6-month follow up and 32 participants at the 12-month follow up met criteria of this subset. Open-ended responses were thematically analyzed."

6b) Any changes to trial outcomes after the trial commenced, with reasons

Does your paper address CONSORT subitem 6b? *

Copy and paste relevant sections from the manuscript (include quotes in quotation marks "like this" to indicate direct quotes from your manuscript), or elaborate on this item by providing additional information not in the ms, or briefly explain why the item is not applicable/relevant for your study

NA no changes to trial outcomes

7a) How sample size was determined

NPT: When applicable, details of whether and how the clustering by care provides or centers was addressed

7a-i) Describe whether and how expected attrition was taken into account when calculating the sample size

Describe whether and how expected attrition was taken into account when calculating the sample size.

	1	2	3	4	5	
subitem not at all important	○	○	○	○	○	essential

Does your paper address subitem 7a-i?

Copy and paste relevant sections from manuscript title (include quotes in quotation marks "like this" to indicate direct quotes from your manuscript), or elaborate on this item by providing additional information not in the ms, or briefly explain why the item is not applicable/relevant for your study

Power calculations, including planned attrition is outlined in the protocol paper "The full study protocol was prospectively published [18]."

7b) When applicable, explanation of any interim analyses and stopping guidelines

Does your paper address CONSORT subitem 7b? *

Copy and paste relevant sections from the manuscript (include quotes in quotation marks "like this" to indicate direct quotes from your manuscript), or elaborate on this item by providing additional information not in the ms, or briefly explain why the item is not applicable/relevant for your study

NA we did not conduct any interim analyses

8a) Method used to generate the random allocation sequence

NPT: When applicable, how care providers were allocated to each trial group

Does your paper address CONSORT subitem 8a? *

Copy and paste relevant sections from the manuscript (include quotes in quotation marks "like this" to indicate direct quotes from your manuscript), or elaborate on this item by providing additional information not in the ms, or briefly explain why the item is not applicable/relevant for your study

"Randomisation

Randomisation of schools was conducted by a biostatistician external to the research team using the blockrand function in R (R Core Team). Schools were stratified according to gender, with the school gender mix consisting of co-educational, predominately male, or predominately female. The threshold for predominately male or predominately female was at >60%. Half of schools were randomly allocated to the Mind your Mate intervention condition (n=6) and half were allocated to the active control condition who received health education as usual (n=6). Teachers, students, and researchers were not blinded to intervention allocation."

8b) Type of randomisation; details of any restriction (such as blocking and block size)

Does your paper address CONSORT subitem 8b? *

Copy and paste relevant sections from the manuscript (include quotes in quotation marks "like this" to indicate direct quotes from your manuscript), or elaborate on this item by providing additional information not in the ms, or briefly explain why the item is not applicable/relevant for your study

"Randomisation

Randomisation of schools was conducted by a biostatistician external to the research team using the blockrand function in R (R Core Team). Schools were stratified according to gender, with the school gender mix consisting of co-educational, predominately male, or predominately female. The threshold for predominately male or predominately female was at >60%. Half of schools were randomly allocated to the Mind your Mate intervention condition (n=6) and half were allocated to the active control condition who received health education as usual (n=6). Teachers, students, and researchers were not blinded to intervention allocation. "

9) Mechanism used to implement the random allocation sequence (such as sequentially numbered containers), describing any steps taken to conceal the sequence until interventions were assigned

Does your paper address CONSORT subitem 9? *

Copy and paste relevant sections from the manuscript (include quotes in quotation marks "like this" to indicate direct quotes from your manuscript), or elaborate on this item by providing additional information not in the ms, or briefly explain why the item is not applicable/relevant for your study

"Randomisation

Randomisation of schools was conducted by a biostatistician external to the research team using the blockrand function in R (R Core Team). Schools were stratified according to gender, with the school gender mix consisting of co-educational, predominately male, or predominately female. The threshold for predominately male or predominately female was at >60%. Half of schools were randomly allocated to the Mind your Mate intervention condition (n=6) and half were allocated to the active control condition who received health education as usual (n=6). Teachers, students, and researchers were not blinded to intervention allocation. "

10) Who generated the random allocation sequence, who enrolled participants, and who assigned participants to interventions

Does your paper address CONSORT subitem 10? *

Copy and paste relevant sections from the manuscript (include quotes in quotation marks "like this" to indicate direct quotes from your manuscript), or elaborate on this item by providing additional information not in the ms, or briefly explain why the item is not applicable/relevant for your study

"Randomisation

Randomisation of schools was conducted by a biostatistician external to the research team using the blockrand function in R (R Core Team). Schools were stratified according to gender, with the school gender mix consisting of co-educational, predominately male, or predominately female. The threshold for predominately male or predominately female was at >60%. Half of schools were randomly allocated to the Mind your Mate intervention condition (n=6) and half were allocated to the active control condition who received health education as usual (n=6). Teachers, students, and researchers were not blinded to intervention allocation."

11a) If done, who was blinded after assignment to interventions (for example, participants, care providers, those assessing outcomes) and how

NPT: Whether or not administering co-interventions were blinded to group assignment

11a-i) Specify who was blinded, and who wasn’t

Specify who was blinded, and who wasn’t. Usually, in web-based trials it is not possible to blind the participants [1, 3] (this should be clearly acknowledged), but it may be possible to blind outcome assessors, those doing data analysis or those administering co-interventions (if any).

	1	2	3	4	5	
subitem not at all important	○	○	○	○	○	essential

Does your paper address subitem 11a-i? *

Copy and paste relevant sections from the manuscript (include quotes in quotation marks "like this" to indicate direct quotes from your manuscript), or elaborate on this item by providing additional information not in the ms, or briefly explain why the item is not applicable/relevant for your study

Participants and researchers were not blinded to allocation.

11a-ii) Discuss e.g., whether participants knew which intervention was the “intervention of interest” and which one was the “comparator”

Informed consent procedures (4a-ii) can create biases and certain expectations - discuss e.g., whether participants knew which intervention was the “intervention of interest” and which one was the “comparator”.

	1	2	3	4	5	
subitem not at all important	○	○	○	○	○	essential

Does your paper address subitem 11a-ii?

Copy and paste relevant sections from the manuscript (include quotes in quotation marks "like this" to indicate direct quotes from your manuscript), or elaborate on this item by providing additional information not in the ms, or briefly explain why the item is not applicable/relevant for your study

Participants were informed about the intervention they would receive as part of the informed consent procedure.

11b) If relevant, description of the similarity of interventions

(this item is usually not relevant for ehealth trials as it refers to similarity of a placebo or sham intervention to a active medication/intervention)

Does your paper address CONSORT subitem 11b? *

Copy and paste relevant sections from the manuscript (include quotes in quotation marks "like this" to indicate direct quotes from your manuscript), or elaborate on this item by providing additional information not in the ms, or briefly explain why the item is not applicable/relevant for your study

NA this was not relevant for this study

12a) Statistical methods used to compare groups for primary and secondary outcomes

NPT: When applicable, details of whether and how the clustering by care providers or centers was addressed

Does your paper address CONSORT subitem 12a? *

Copy and paste relevant sections from the manuscript (include quotes in quotation marks "like this" to indicate direct quotes from your manuscript), or elaborate on this item by providing additional information not in the ms, or briefly explain why the item is not applicable/relevant for your study

"Statistical Analysis
As per our preregistered analysis plan, multilevel mixed effect regression models were used to determine the effects of the intervention in Stata (version 17.0; StataCorp) [27,28]. The mixed effect repeated measures approach examines change over time both within and between groups while accounting for baseline differences and attrition. The intervention effects over time are modeled by a group-by-time interaction, where time is coded as a continuous linear variable representing baseline and the 6-month and 12-month follow-up assessments. The intraclass correlation showed that less than 10% of the variance existed at the school level; thus, 3-level models were abandoned instead of 2-level models for all outcomes [29]. All models included a random intercept at the individual level to account for the correlation between repeat measures over time, and a random slope was added when it improved the model fit, and the best-fitting covariance structure was tested using likelihood ratio tests, including the Akaike Information Criterion [30].
Time was coded as a continuous linear variable representing baseline and the 6-month and 12-month follow-up assessments. The time main effect represents the estimated 1-year change in the outcome in the control group over time, and the group main effect indicates differences in dependent variables between the intervention group and the control at baseline (referent category was the control group). To address the primary aims of the trial, intervention efficacy is modeled using a group-by-time interaction, representing the relative change in odds (logistic outcomes: alcohol use, drug use, help-seeking intentions, and help-seeking behavior) or mean frequency (continuous outcomes: knowledge [literacy], depression, psychological distress, generalized anxiety, and help seeking) in each outcome over 12 months. The interaction term for the binary outcomes was modeled using the logit link function, and the odds were calculated by exponentiating the regression coefficient. All continuous dependent outcomes were modeled using multilevel effect analysis. The assumption of normality was examined and sensitivity analyses with transformed data were conducted when the normality assumptions were violated. Full information maximum likelihood procedures were used to handle missing data, whereby all available data were included in the model estimates under the assumption that data were missing at random, and analyses were conducted on the intention-to-treat sample."

12a-i) Imputation techniques to deal with attrition / missing values

Imputation techniques to deal with attrition / missing values: Not all participants will use the intervention/comparator as intended and attrition is typically high in ehealth trials. Specify how participants who did not use the application or dropped out from the trial were treated in the statistical analysis (a complete case analysis is strongly discouraged, and simple imputation techniques such as LOCF may also be problematic [4]).

	1	2	3	4	5	
subitem not at all important	○	○	○	○	○	essential

Does your paper address subitem 12a-i? *

Copy and paste relevant sections from the manuscript (include quotes in quotation marks "like this" to indicate direct quotes from your manuscript), or elaborate on this item by providing additional information not in the ms, or briefly explain why the item is not applicable/relevant for your study

"Full information
maximum likelihood procedures were used to handle missing
data, whereby all available data were included in the model
estimates under the assumption that data were missing at
random, and analyses were conducted on the intention-to-treat
sample."

12b) Methods for additional analyses, such as subgroup analyses and adjusted analyses

Does your paper address CONSORT subitem 12b? *

Copy and paste relevant sections from the manuscript (include quotes in quotation marks "like this" to indicate direct quotes from your manuscript), or elaborate on this item by providing additional information not in the ms, or briefly explain why the item is not applicable/relevant for your study

"The assumption of normality was examined and
sensitivity analyses with transformed data were conducted when
the normality assumptions were violated."

X26) REB/IRB Approval and Ethical Considerations [recommended as subheading under "Methods"] (not a CONSORT item)

X26-i) Comment on ethics committee approval

	1	2	3	4	5	
subitem not at all important	○	○	○	○	○	essential

Does your paper address subitem X26-i?

Copy and paste relevant sections from the manuscript (include quotes in quotation marks "like this" to indicate direct quotes from your manuscript), or elaborate on this item by providing additional information not in the ms, or briefly explain why the item is not applicable/relevant for your study

"Ethics Approval
This study was approved by the University of Sydney Human
Research Ethics Committee, Australia (project number:
2020/054), and prospectively registered in the Australian New
Zealand Clinical Trials Registry (ACTRN 12620000753954)."

"Access to schools was granted by the New South Wales (NSW)
Department of Education and Communities State Education
Research Applications Process (2020130)."

x26-ii) Outline informed consent procedures

Outline informed consent procedures e.g., if consent was obtained offline or online (how? Checkbox, etc.?), and what information was provided (see 4a-ii). See [6] for some items to be included in informed consent documents.

	1	2	3	4	5	
subitem not at all important	○	○	○	○	○	essential

Does your paper address subitem X26-ii?

Copy and paste relevant sections from the manuscript (include quotes in quotation marks "like this" to indicate direct quotes from your manuscript), or elaborate on this item by providing additional information not in the ms, or briefly explain why the item is not applicable/relevant for your study

"Opt-in parental and guardian information and consent forms were sent electronically or via hard copy to all parents or guardians of the selected year group. Students were eligible to participate in the trial if they received parental consent, provided active consent, and owned a smartphone."

X26-iii) Safety and security procedures

Safety and security procedures, incl. privacy considerations, and any steps taken to reduce the likelihood or detection of harm (e.g., education and training, availability of a hotline)

	1	2	3	4	5	
subitem not at all important	○	○	○	○	○	essential

Does your paper address subitem X26-iii?

Copy and paste relevant sections from the manuscript (include quotes in quotation marks "like this" to indicate direct quotes from your manuscript), or elaborate on this item by providing additional information not in the ms, or briefly explain why the item is not applicable/relevant for your study

"Throughout the app use,
it was emphasized that students were not responsible for their
friends and their friends' choices and were encouraged to
connect their peers with a responsible adult. Crisis helpline
numbers were easily accessible on all screens and under "support
options."

RESULTS

13a) For each group, the numbers of participants who were randomly assigned, received intended treatment, and were analysed for the primary outcome

NPT: The number of care providers or centers performing the intervention in each group and the number of patients treated by each care provider in each center

Does your paper address CONSORT subitem 13a? *

Copy and paste relevant sections from the manuscript (include quotes in quotation marks "like this" to indicate direct quotes from your manuscript), or elaborate on this item by providing additional information not in the ms, or briefly explain why the item is not applicable/relevant for your study

This information is contained in Table 1 and Figure 1.

13b) For each group, losses and exclusions after randomisation, together with reasons

Does your paper address CONSORT subitem 13b? (NOTE: Preferably, this is shown in a CONSORT flow diagram) *

Copy and paste relevant sections from the manuscript (include quotes in quotation marks "like this" to indicate direct quotes from your manuscript), or elaborate on this item by providing additional information not in the ms, or briefly explain why the item is not applicable/relevant for your study

This information is contained in Figure 1.

13b-i) Attrition diagram

Strongly recommended: An attrition diagram (e.g., proportion of participants still logging in or using the intervention/comparator in each group plotted over time, similar to a survival curve) or other figures or tables demonstrating usage/dose/engagement.

	1	2	3	4	5	
subitem not at all important	○	○	○	○	○	essential

Does your paper address subitem 13b-i?

Copy and paste relevant sections from the manuscript or cite the figure number if applicable (include quotes in quotation marks "like this" to indicate direct quotes from your manuscript), or elaborate on this item by providing additional information not in the ms, or briefly explain why the item is not applicable/relevant for your study

This information is contained in Figure 1.

14a) Dates defining the periods of recruitment and follow-up

Does your paper address CONSORT subitem 14a? *

Copy and paste relevant sections from the manuscript (include quotes in quotation marks "like this" to indicate direct quotes from your manuscript), or elaborate on this item by providing additional information not in the ms, or briefly explain why the item is not applicable/relevant for your study

"Recruitment took place between June 2020 and March 2021."

"Baseline data collection was conducted between November 2020 and May 2021, with students completing follow-up surveys at 6 and 12 months after baseline data collection (final follow-up data were collected by May 2022)."

14a-i) Indicate if critical “secular events” fell into the study period

Indicate if critical “secular events” fell into the study period, e.g., significant changes in Internet resources available or “changes in computer hardware or Internet delivery resources”

	1	2	3	4	5	
subitem not at all important	○	○	○	○	○	essential

Does your paper address subitem 14a-i?

Copy and paste relevant sections from the manuscript (include quotes in quotation marks "like this" to indicate direct quotes from your manuscript), or elaborate on this item by providing additional information not in the ms, or briefly explain why the item is not applicable/relevant for your study

"It is also noteworthy that the trial was conducted during the initial years of the COVID-19 pandemic from November 2020 to May 2022, a time of unprecedented change and stress for adolescents who were transitioning in and out of home schooling and were facing limited interaction with peers."

14b) Why the trial ended or was stopped (early)

Does your paper address CONSORT subitem 14b? *

Copy and paste relevant sections from the manuscript (include quotes in quotation marks "like this" to indicate direct quotes from your manuscript), or elaborate on this item by providing additional information not in the ms, or briefly explain why the item is not applicable/relevant for your study

NA the trial was not ended or stopped early

15) A table showing baseline demographic and clinical characteristics for each group

NPT: When applicable, a description of care providers (case volume, qualification, expertise, etc.) and centers (volume) in each group

Does your paper address CONSORT subitem 15? *

Copy and paste relevant sections from the manuscript (include quotes in quotation marks "like this" to indicate direct quotes from your manuscript), or elaborate on this item by providing additional information not in the ms, or briefly explain why the item is not applicable/relevant for your study

This information is contained in Table 1

15-i) Report demographics associated with digital divide issues

In ehealth trials it is particularly important to report demographics associated with digital divide issues, such as age, education, gender, social-economic status, computer/Internet /ehealth literacy of the participants, if known.

	1	2	3	4	5	
subitem not at all important	○	○	○	○	○	essential

Does your paper address subitem 15-i? *

Copy and paste relevant sections from the manuscript (include quotes in quotation marks "like this" to indicate direct quotes from your manuscript), or elaborate on this item by providing additional information not in the ms, or briefly explain why the item is not applicable/relevant for your study

This information is contained in Table 1.

16) For each group, number of participants (denominator) included in each analysis and whether the analysis was by original assigned groups

16-i) Report multiple “denominators” and provide definitions

Report multiple “denominators” and provide definitions: Report N’s (and effect sizes) “across a range of study participation [and use] thresholds” [1], e.g., N exposed, N consented, N used more than x times, N used more than y weeks, N participants “used” the intervention/comparator at specific pre-defined time points of interest (in absolute and relative numbers per group). Always clearly define “use” of the intervention.

	1	2	3	4	5	
subitem not at all important	○	○	○	○	○	essential

Does your paper address subitem 16-i? *

Copy and paste relevant sections from the manuscript (include quotes in quotation marks "like this" to indicate direct quotes from your manuscript), or elaborate on this item by providing additional information not in the ms, or briefly explain why the item is not applicable/relevant for your study

"Full information
maximum likelihood procedures were used to handle missing
data, whereby all available data were included in the model
estimates under the assumption that data were missing at
random, and analyses were conducted on the intention-to-treat
sample."

16-ii) Primary analysis should be intent-to-treat

Primary analysis should be intent-to-treat, secondary analyses could include comparing only “users”, with the appropriate caveats that this is no longer a randomized sample (see 18-i).

	1	2	3	4	5	
subitem not at all important	○	○	○	○	○	essential

Does your paper address subitem 16-ii?

Copy and paste relevant sections from the manuscript (include quotes in quotation marks "like this" to indicate direct quotes from your manuscript), or elaborate on this item by providing additional information not in the ms, or briefly explain why the item is not applicable/relevant for your study

"Full information
maximum likelihood procedures were used to handle missing
data, whereby all available data were included in the model
estimates under the assumption that data were missing at
random, and analyses were conducted on the intention-to-treat
sample."

17a) For each primary and secondary outcome, results for each group, and the estimated effect size and its precision (such as 95% confidence interval)

Does your paper address CONSORT subitem 17a? *

Copy and paste relevant sections from the manuscript (include quotes in quotation marks "like this" to indicate direct quotes from your manuscript), or elaborate on this item by providing additional information not in the ms, or briefly explain why the item is not applicable/relevant for your study

This information is contained in Table 3 and Table 4

17a-i) Presentation of process outcomes such as metrics of use and intensity of use

In addition to primary/secondary (clinical) outcomes, the presentation of process outcomes such as metrics of use and intensity of use (dose, exposure) and their operational definitions is critical. This does not only refer to metrics of attrition (13-b) (often a binary variable), but also to more continuous exposure metrics such as “average session length”. These must be accompanied by a technical description how a metric like a “session” is defined (e.g., timeout after idle time) [1] (report under item 6a).

	1	2	3	4	5	
subitem not at all important	○	○	○	○	○	essential

Does your paper address subitem 17a-i?

Copy and paste relevant sections from the manuscript (include quotes in quotation marks "like this" to indicate direct quotes from your manuscript), or elaborate on this item by providing additional information not in the ms, or briefly explain why the item is not applicable/relevant for your study

"Acceptability
Acceptability analysis measured a subset of intervention participants who met the following criteria: (1) those who completed the app evaluation section of the surveys and (2) those who indicated that they downloaded the app. A total of 25 participants at the 6-month follow-up and 32 participants at the 12-month follow-up met the criteria for this subset. The open-ended responses were thematically analyzed. At the 6-month follow-up, 36% (9/25) of students had used the app, and 20% (5/25) reported using it once or twice; at the 12-month follow-up, this increased to 50% (16/32) of students having used the app and 41% (13/32) having used it once or twice. Students who at least "slightly agreed" that they liked the Mind your Mate app accounted for 48% (12/25) and 44% (11/25) of this subset at 6-month and 12-month follow-ups, respectively. Throughout the intervention, the average 5-star rating of the app remained stable from 3.48 to 3.38 stars (SD 0.57 and 0.82 at 6-month and 12-month follow-ups, respectively). Most students indicated that "maybe" there are "several people" to whom they would recommend the app. When asked about the likelihood of the app increasing their mental health and well-being awareness, those who at least "slightly agreed" made up 56% (14/25) at the 6-month and 12-month follow-ups. Regarding the app's usefulness, nearly half of the students agreed that it was useful, whereas the remaining half were "undecided" at both follow-up occasions. Multiple open-ended comments stated that the app was "useful" and "helpful." At the 6-month and 12-month follow-ups, about half of the students "slightly agree" to "strongly agree" that the app is likely to encourage further help seeking.
In a separate subset of students who did not download the app, open-ended responses regarding why they did not download the app were thematically analyzed. A total of 15 responses were captured at the 6-month follow-up and 20 responses at the 12-month follow-up. Participants' reasons for not downloading the app were as follows, from most to least frequent: no motivation (n=3 and 5 at 6-month and 12-month follow-ups, respectively), not useful (n=2 and 6 at 6-month and 12-month follow-ups, respectively), forgot to download it (n=2 and 3 at 6-month and 12-month follow-ups, respectively), high storage requirement (n=2 and 3 at 6-month and 12-month follow-ups, respectively), missed the lesson with download instructions (n=2 and 2 at 6-month and 12-month follow-ups, respectively), no time to use the app (n=1 and 1 at 6-month and 12-month follow-ups, respectively), no one to connect to (n=1 and 1 at 6-month and 12-month follow-ups, respectively), and privacy concerns (n=1 and 0 at 6-month and 12-month follow-ups, [illegible]

respectively). The full details of the responses can be found in Multimedia Appendix 1."

17b) For binary outcomes, presentation of both absolute and relative effect sizes is recommended

Does your paper address CONSORT subitem 17b? *

Copy and paste relevant sections from the manuscript (include quotes in quotation marks "like this" to indicate direct quotes from your manuscript), or elaborate on this item by providing additional information not in the ms, or briefly explain why the item is not applicable/relevant for your study

This information is contained in Table 4.

18) Results of any other analyses performed, including subgroup analyses and adjusted analyses, distinguishing pre-specified from exploratory

Does your paper address CONSORT subitem 18? *

Copy and paste relevant sections from the manuscript (include quotes in quotation marks "like this" to indicate direct quotes from your manuscript), or elaborate on this item by providing additional information not in the ms, or briefly explain why the item is not applicable/relevant for your study

This information is contained in the multimedia appendix

18-i) Subgroup analysis of comparing only users

A subgroup analysis of comparing only users is not uncommon in ehealth trials, but if done, it must be stressed that this is a self-selected sample and no longer an unbiased sample from a randomized trial (see 16-iii).

	1	2	3	4	5	
subitem not at all important	○	○	○	○	○	essential

Does your paper address subitem 18-i?

Copy and paste relevant sections from the manuscript (include quotes in quotation marks "like this" to indicate direct quotes from your manuscript), or elaborate on this item by providing additional information not in the ms, or briefly explain why the item is not applicable/relevant for your study

This subgroup analyses was not conducted in the current trial.

19) All important harms or unintended effects in each group

(for specific guidance see CONSORT for harms)

Does your paper address CONSORT subitem 19? *

Copy and paste relevant sections from the manuscript (include quotes in quotation marks "like this" to indicate direct quotes from your manuscript), or elaborate on this item by providing additional information not in the ms, or briefly explain why the item is not applicable/relevant for your study

All effects are reported in Table 3 and Table 4.

19-i) Include privacy breaches, technical problems

Include privacy breaches, technical problems. This does not only include physical “harm” to participants, but also incidents such as perceived or real privacy breaches [1], technical problems, and other unexpected/unintended incidents. “Unintended effects” also includes unintended positive effects [2].

	1	2	3	4	5	
subitem not at all important	○	○	○	○	○	essential

Does your paper address subitem 19-i?

Copy and paste relevant sections from the manuscript (include quotes in quotation marks "like this" to indicate direct quotes from your manuscript), or elaborate on this item by providing additional information not in the ms, or briefly explain why the item is not applicable/relevant for your study

We did not encounter privacy breaches or other significant technical problems.

19-ii) Include qualitative feedback from participants or observations from staff/researchers

Include qualitative feedback from participants or observations from staff/researchers, if available, on strengths and shortcomings of the application, especially if they point to unintended/unexpected effects or uses. This includes (if available) reasons for why people did or did not use the application as intended by the developers.

	1	2	3	4	5	
subitem not at all important	○	○	○	○	○	essential

Does your paper address subitem 19-ii?

Copy and paste relevant sections from the manuscript (include quotes in quotation marks "like this" to indicate direct quotes from your manuscript), or elaborate on this item by providing additional information not in the ms, or briefly explain why the item is not applicable/relevant for your study

This information is contained in the multimedia appendix.

DISCUSSION

22) Interpretation consistent with results, balancing benefits and harms, and considering other relevant evidence

NPT: In addition, take into account the choice of the comparator, lack of or partial blinding, and unequal expertise of care providers or centers in each group

22-i) Restate study questions and summarize the answers suggested by the data, starting with primary outcomes and process outcomes (use)

Restate study questions and summarize the answers suggested by the data, starting with primary outcomes and process outcomes (use).

	1	2	3	4	5	
subitem not at all important	○	○	○	○	○	essential

Does your paper address subitem 22-i? *

Copy and paste relevant sections from the manuscript (include quotes in quotation marks "like this" to indicate direct quotes from your manuscript), or elaborate on this item by providing additional information not in the ms, or briefly explain why the item is not applicable/relevant for your study

"The program aimed to foster peer support among adolescents using
digital technology. Mind your Mate was found to result in reductions in depressive symptoms among students in the
intervention group up to 12 months after baseline when
compared with a control condition, and the size of this effect
was small to moderate. No differences were observed between
the intervention and control group in relation to all other
outcomes (ie, mental health and substance use literacy, anxiety
symptoms, substance use, and help seeking)."

22-ii) Highlight unanswered new questions, suggest future research

Highlight unanswered new questions, suggest future research.

	1	2	3	4	5	
subitem not at all important	○	○	○	○	○	essential

Does your paper address subitem 22-ii?

Copy and paste relevant sections from the manuscript (include quotes in quotation marks "like this" to indicate direct quotes from your manuscript), or elaborate on this item by providing additional information not in the ms, or briefly explain why the item is not applicable/relevant for your study

"Future research using the app may
also benefit from the inclusion of further qualitative data
collection to better understand how the app made an impact,
including interviews with the adolescents and possibly their
parents."

20) Trial limitations, addressing sources of potential bias, imprecision, and, if relevant, multiplicity of analyses

20-i) Typical limitations in ehealth trials

Typical limitations in ehealth trials: Participants in ehealth trials are rarely blinded. Ehealth trials often look at a multiplicity of outcomes, increasing risk for a Type I error. Discuss biases due to non-use of the intervention/usability issues, biases through informed consent procedures, unexpected events.

	1	2	3	4	5	
subitem not at all important	○	○	○	○	○	essential

Does your paper address subitem 20-i? *

Copy and paste relevant sections from the manuscript (include quotes in quotation marks "like this" to indicate direct quotes from your manuscript), or elaborate on this item by providing additional information not in the ms, or briefly explain why the item is not applicable/relevant for your study

"Despite these strengths,
our findings must be considered in light of several limitations. Although we were able to recruit our target number of schools, student participation within the schools was lower than anticipated, largely because of a lack of return of active parental consent forms. Parental consent was heavily impacted by the COVID-19 pandemic, which placed extreme pressure on school staff and parents during the time in which consent forms were distributed for this study. Although the research team worked closely with schools to implement a range of strategies including multiple forms of consent (eg, electronic and hard copy), reminder emails, and articles in school newsletters, the resulting consent rate was reflective of the challenging time in which consent for the research study was conducted. Importantly, although consent was required to participate in the surveys, all students at the participating schools received the intervention component as it mapped onto their existing curriculum. The research team also worked closely with schools to be flexible at the start of the study, as recruitment took longer than anticipated owing to disruptions in school closures because of the COVID-19 pandemic. It is acknowledged that analyses may be underpowered to detect significant effects on all outcomes, and replication in a larger-scale RCT is needed to confirm the efficacy of the program."

21) Generalisability (external validity, applicability) of the trial findings

NPT: External validity of the trial findings according to the intervention, comparators, patients, and care providers or centers involved in the trial

21-i) Generalizability to other populations

Generalizability to other populations: In particular, discuss generalizability to a general Internet population, outside of a RCT setting, and general patient population, including applicability of the study results for other organizations

	1	2	3	4	5	
subitem not at all important	○	○	○	○	○	essential

Does your paper address subitem 21-i?

Copy and paste relevant sections from the manuscript (include quotes in quotation marks "like this" to indicate direct quotes from your manuscript), or elaborate on this item by providing additional information not in the ms, or briefly explain why the item is not applicable/relevant for your study

"Key strengths of this study include the use of a rigorous randomized controlled design with preregistration of outcomes and hypotheses [18] and the inclusion of students from both public and private schools."

21-ii) Discuss if there were elements in the RCT that would be different in a routine application setting

Discuss if there were elements in the RCT that would be different in a routine application setting (e.g., prompts/reminders, more human involvement, training sessions or other co-interventions) and what impact the omission of these elements could have on use, adoption, or outcomes if the intervention is applied outside of a RCT setting.

	1	2	3	4	5	
subitem not at all important	○	○	○	○	○	essential

Does your paper address subitem 21-ii?

Copy and paste relevant sections from the manuscript (include quotes in quotation marks "like this" to indicate direct quotes from your manuscript), or elaborate on this item by providing additional information not in the ms, or briefly explain why the item is not applicable/relevant for your study

The study was conducted within the school setting and this is discussed in the manuscript.

OTHER INFORMATION

23) Registration number and name of trial registry

Does your paper address CONSORT subitem 23? *

Copy and paste relevant sections from the manuscript (include quotes in quotation marks "like this" to indicate direct quotes from your manuscript), or elaborate on this item by providing additional information not in the ms, or briefly explain why the item is not applicable/relevant for your study

"Trial Registration: Australian New Zealand Clinical Trials Registry (ANZCTR) ACTRN12620000753954;
https://anzctr.org.au/Trial/Registration/TrialReview.aspx?ACTRN=12620000753954
International Registered Report Identifier (IRRID): RR2-10.2196/26796"

24) Where the full trial protocol can be accessed, if available

Does your paper address CONSORT subitem 24? *

Cite a Multimedia Appendix, other reference, or copy and paste relevant sections from the manuscript (include quotes in quotation marks "like this" to indicate direct quotes from your manuscript), or elaborate on this item by providing additional information not in the ms, or briefly explain why the item is not applicable/relevant for your study

"The full study protocol including the initial
sample size calculations was prospectively published, and it
was estimated that 12 schools with at least 720 students would
be required to achieve 80% power to detect an effect size of 0.2
[18]."

25) Sources of funding and other support (such as supply of drugs), role of funders

Does your paper address CONSORT subitem 25? *

Copy and paste relevant sections from the manuscript (include quotes in quotation marks "like this" to indicate direct quotes from your manuscript), or elaborate on this item by providing additional information not in the ms, or briefly explain why the item is not applicable/relevant for your study

"LB is funded by an Australian Rotary Health Bruce Edwards Postdoctoral Research Fellowship. Some of the development costs for
the Mind your Mate intervention came from a seed funding grant from the Brain and Mind Centre, the University of Sydney."

X27) Conflicts of Interest (not a CONSORT item)

X27-i) State the relation of the study team towards the system being evaluated

In addition to the usual declaration of interests (financial or otherwise), also state the relation of the study team towards the system being evaluated, i.e., state if the authors/evaluators are distinct from or identical with the developers/sponsors of the intervention.

	1	2	3	4	5	
subitem not at all important	○	○	○	○	○	essential

Does your paper address subitem X27-i?

Copy and paste relevant sections from the manuscript (include quotes in quotation marks "like this" to indicate direct quotes from your manuscript), or elaborate on this item by providing additional information not in the ms, or briefly explain why the item is not applicable/relevant for your study

"Conflicts of Interest
LB developed the Mind your Mate program."

About the CONSORT EHEALTH checklist

As a result of using this checklist, did you make changes in your manuscript? *

- ○ yes, major changes
- ○ yes, minor changes
- ◉ no

What were the most important changes you made as a result of using this checklist?

Your answer

How much time did you spend on going through the checklist INCLUDING making changes in your manuscript *

Aprox 5 hours on the checklist

As a result of using this checklist, do you think your manuscript has improved? *

- (x) yes
- ( ) no
- ( ) Other:

Would you like to become involved in the CONSORT EHEALTH group?

This would involve for example becoming involved in participating in a workshop and writing an "Explanation and Elaboration" document

- ( ) yes
- (x) no
- ( ) Other:

Clear selection

Any other comments or questions on CONSORT EHEALTH

Your answer

STOP - Save this form as PDF before you click submit
To generate a record that you filled in this form, we recommend to generate a PDF of this page (on a Mac, simply select "print" and then select "print as PDF") before you submit it.

When you submit your (revised) paper to JMIR, please upload the PDF as supplementary file.

Don't worry if some text in the textboxes is cut off, as we still have the complete information in our database. Thank you!

Final step: Click submit !
Click submit so we have your answers in our database!

Submit

Clear form

Never submit passwords through Google Forms.

This content is neither created nor endorsed by Google. Report Abuse - Terms of Service - Privacy Policy

Google Forms
